# In Vitro Gastrointestinal Digestion of *Calanus finmarchicus* Products: Amino Acid Composition, Degree of Hydrolysis, Antioxidant Capacity, and Antidiabetic Activity

**DOI:** 10.3390/md24070240

**Published:** 2026-07-07

**Authors:** Ying Wang, Karl-Erik Eilertsen, Edel Oddny Elvevoll, Chun Li, Ida-Johanne Jensen

**Affiliations:** 1 Faculty of Biosciences, Fisheries and Economics, The Norwegian College of Fishery Science, UiT-The Arctic University of Norway, 9037 Tromsø, Norway; karl-erik.eilertsen@uit.no (K.-E.E.); edel.elvevoll@uit.no (E.O.E.); chun.li@uit.no (C.L.); idaj.jensen@ntnu.no (I.-J.J.); 2 Department of Biotechnology and Food Science, Norwegian University of Science and Technology, NTNU, 7491 Trondheim, Norway

**Keywords:** *Calanus finmarchicus*, hydrolysate, antioxidant capacity, antidiabetic activity, nutraceutical, marine crustacean, DPP-IV, PTP1B, FRAP, ORAC

## Abstract

Marine rest raw materials are often undervalued or wasted despite their nutrient and bioactive composition. *Calanus finmarchicus*, harvested primarily for its omega-3-rich oil, yields a side-stream protein hydrolysate, *C. finmarchicus* hydrolysate (CFH), during commercial enzyme-assisted extraction. Although currently used as a feed ingredient, CFH contains low-molecular-weight peptides and free amino acids with potential for human health applications. This study evaluated the gastrointestinal stability of CFH and the impact of digestion on bioactivity using a static in vitro gastrointestinal digestion model. Fresh-frozen and freeze-dried *C. finmarchicus* were included to provide comparative data. Antioxidant capacity was measured by ferric reducing antioxidant power (FRAP) and oxygen radical absorbance capacity (ORAC) assays, and antidiabetic activity by dipeptidyl peptidase-IV (DPP-IV) and protein tyrosine phosphatase 1B (PTP1B) inhibition assays. The hydrolysate maintained its antioxidant capacity throughout digestion (at 165 min: FRAP: 27.5 ± 0.6 µmol TE/g dry weight (DW); ORAC: 411 ± 37 µmol TE/g DW). Digestion increased its DPP-IV inhibitory activity, with the inhibitory concentration (IC_50_) decreased from 3.73 to 1.96 mg/mL (*p* ≥ 0.05). PTP1B inhibitors were nonselective and detected only at 0 and 30 min. These findings support our hypothesis that CFH may serve as a nutraceutical for humans and provide a rationale for subsequent in vivo studies. However, further identification of bioactive components and in vivo validation are warranted.

## 1. Introduction

The marine environment, covering approximately 71% of the Earth’s surface, is a vast reservoir of natural resources. Marine organisms, including fish and other seafood, are recognized as rich sources of essential nutrients such as high-quality proteins, long-chain omega-3 polyunsaturated fatty acids (LC n-3 PUFAs), vitamins A and D, and bioactive compounds with potential health benefits [1,2]. Global fisheries and aquaculture production continues to grow due to population growth and consumer preferences [3]. The expansion of fishery and aquaculture production has also generated a significant increase in side-stream products, which often contain valuable nutrients yet are treated as low-value materials or wastes. Recent research has highlighted the potential for valorizing marine rest raw materials into value-added products, such as protein hydrolysates and bioactive peptides [4]. Protein hydrolysates recovered from marine side-streams have exhibited a wide range of bioactive properties, particularly antioxidant and antidiabetic activities, making them promising candidates for alleviating metabolic disorders, such as oxidative stress and hyperglycemia.

During normal metabolic processes in living cells, unstable oxygen-containing molecules known as reactive oxygen species (ROS) are generated. Under normal physiological conditions, the antioxidant defense system in the human body can eliminate generated ROS and maintain redox homeostasis. However, under abnormal physiological conditions, the production of ROS exceeds the buffering capacity of the endogenous antioxidants, resulting in ROS accumulation. Prolonged and excessive accumulation of ROS can break redox homeostasis and induce oxidative stress, which is linked to cellular dysfunctions and chronic diseases like type-2 diabetes (T2D), raising substantial concerns for human health [5].

Exogenous antioxidants such as antioxidative peptides and amino acids may help neutralize ROS and restore redox homeostasis, mitigating the damaging effects induced by oxidative stress and ameliorating the development of T2D [5]. Widely recognized therapeutic targets for T2D include dipeptidyl peptidase-IV (DPP-IV) and protein tyrosine phosphatase 1B (PTP1B), whose inhibition may help maintain blood glucose homeostasis and modulate insulin sensitivity [6].

Several marine crustaceans, namely shrimp, krill, and crab, and their protein hydrolysates have been reported to exhibit antioxidant, anti-inflammatory, and antidiabetic activities [7,8]. *Calanus finmarchicus* (Gunnerus, 1770), a small crustacean abundant in the Northern Atlantic and Arctic Oceans, has emerged as a novel low-trophic marine resource [9]. The species has a total annual reproduction rate of around 290 million tonnes and an estimated standing biomass of 33 million tonnes in the Norwegian Sea, supporting an ecologically safe annual quota of 254 thousand tonnes [10,11]. However, the current harvest accounts for only 1% of this quota due to low market demand [11]. *C. finmarchicus* is primarily harvested for its marine oil rich in wax esters and LC n-3 PUFAs [12]. During the enzyme-assisted commercial oil extraction process, a liquid hydrolysate side-stream is recovered. The protein fraction of this *Calanus* hydrolysate (hereafter referred to as CFH), consisting of approximately 87% low-molecular-weight peptides (≤1000 Da) and free amino acids, has demonstrated in vitro antioxidant and antidiabetic activities [13,14]. However, these bioactive peptides may be further hydrolyzed during gastrointestinal digestion, potentially reducing their efficacy before reaching the bloodstream or target cells. Therefore, it is important to characterize their gastrointestinal stability. To our knowledge, most studies on CFH have focused on its potential as a feed ingredient, with comparatively little attention to human health applications.

The primary aim of this study was to evaluate the gastrointestinal stability of CFH and the effects of digestion on its antioxidant and antidiabetic activities using a static in vitro gastrointestinal digestion model. Fresh-frozen *C. finmarchicus* (FFCF) and freeze-dried *C. finmarchicus* (FDCF) were included to provide comparative data on proximate composition, amino acid composition, bioactivity, and bioaccessibility, and to deepen the understanding of this zooplankton. We hypothesized that CFH contains low-molecular-weight peptides that remain stable during digestion and retain antioxidant and antidiabetic activities. In contrast, FFCF and FDCF were expected to release additional peptides during digestion, potentially altering overall bioactivity. Gastrointestinal stability was assessed by monitoring the release of free amino acids (FAA) and the degree of hydrolysis (DH). Antioxidant capacity was measured using the ferric reducing antioxidant power (FRAP) and oxygen radical absorbance capacity (ORAC) assays, whereas antidiabetic potential was evaluated by screening for DPP-IV and PTP1B inhibitors. To our knowledge, this is the first comparative in vitro digestion study across *C. finmarchicus* fresh-frozen biomass, freeze-dried biomass, and industrial hydrolysate, directly linking digestion dynamics to antioxidant capacity and antidiabetic activity. Our results indicate that no additional release of primary amino groups or FAA from CFH occurred during gastrointestinal digestion. Consistent with this, its FRAP and ORAC antioxidant capacities were maintained, and its DPP-IV inhibitory activity was insignificantly enhanced throughout digestion. In contrast, the nonselective PTP1B inhibitory activity was detectable only in the initial phases of digestion. These findings support our hypothesis that the bioactive peptides in CFH remain stable during digestion, highlighting the potential to valorize CFH as a value-added food ingredient or nutraceutical.

## 2. Results and Discussion

### 2.1. Proximate Composition of Undigested Materials

As a liquid hydrolysate, CFH contained 53.7% moisture; its native biomasses, FFCF and FDCF, comprised 87.3% and 12.0% moisture, respectively (Table 1). On a dry weight (DW) basis, protein content was highest in CFH (43.4%) compared to FFCF (36.0%) and FDCF (40.3%). In addition, CFH contained significantly lower lipid (3.8% DW) than FFCF (20.1% DW) and FDCF (17.8% DW).

The moisture content of CFH is consistent with previously reported values (47–64%) [15,16]. However, its protein content is slightly lower compared to previously reported values (47–63% DW) [15,16]. The measured ash and lipid contents of FFCF align with published results, whereas the protein content, at 4.7% on a wet weight (WW) basis, is lower than the values measured using the Kjeldahl method (11.0% WW and 7.6% WW) [13,17].

Given the high proteolytic activity and postmortem autolysis in *C. finmarchicus*, freeze-drying has been recommended as a suitable method for dehydration [18]. The cited study also noted slow moisture removal, with a residual moisture content of 2% after 100 h of vacuum freeze-drying. In line with this, our FDCF samples retained 12.0% moisture after 72 h of vacuum freeze-drying. The proximate composition of FDCF is sparsely reported in the literature, limiting cross-study comparisons.

**Table 1:** Proximate composition of fresh-frozen *C. finmarchicus* (FFCF), freeze-dried *C. finmarchicus* (FDCF), and *C. finmarchicus* hydrolysate (CFH) on a wet weight (WW) and a dry weight (DW) basis. Results are the mean ± standard deviation (SD), with *n* as the number of replicates. Superscript letters within the same column indicate significant differences based on one-way ANOVA followed by Tukey’s post hoc test (*p* < 0.05).

	Water (g/100 g)	*n*	Ash (g/100 g)		*n*	Lipid (g/100 g)		*n*	Protein (g/100 g)		*n*
	WW		WW	DW		WW	DW		WW	DW	
FFCF	87.3 ± 0.5 ^1^	9	2.5 ± 0.1 ^1^	20.0 ± 1.6 ^b^	9	2.6 ± 0.3 ^1^	20.1 ± 2.4 ^a^	6	4.7 ± 0.3	36.0 ± 2.6 ^b^	3
FDCF	12.0 ± 0.1 ^1^	5	17.8 ± 0.1 ^1^	20.2 ± 0.1 ^b^	5	15.7 ± 0.3 ^1^	17.8 ± 0.3 ^a^		35.5 ± 1.3	40.3 ± 1.4 ^a^	
CFH	53.7 ± 0.0		12.4 ± 0.0	26.7 ± 0.1 ^a^		1.8 ± 0.5	3.8 ± 1.0 ^b^		19.9 ± 0.8	43.3 ± 1.8 ^a^	

^1^ Published data [19].

Proteins are essential for growth and maintenance and are therefore key components for human and animal diets. For a healthy adult, the recommended protein intake is 0.83 g per kg body weight per day, which equals 54 g of protein per day for a 65 kg adult [20]. Seafood is generally regarded as a high-quality protein source, with 150 g of edible seafood supplying approximately 50% of this daily requirement [21,22]. The protein content in FFCF is lower than reported values for Antarctic krill (17.2 g/100 g WW) [23], Atlantic salmon fillet (18.8 g/100 g WW) [24], and several shrimp meats (12.3–15.1 g/100 g WW) [25]. By contrast, FDCF and CFH contain substantially higher protein levels and could serve as potential supplemental protein sources for humans and animals.

In general, the proximate composition of *C. finmarchicus* is influenced by endogenous factors (e.g., prey composition and life-cycle stage) and exogenous factors (e.g., sampling location and timing and preservation conditions). Variations in the moisture content of commercially recovered CFH likely reflect processing differences, particularly the final evaporation step [15]. Between-studies differences in protein content likely reflect both natural variation in protein deposition in the native biomass and the methodological differences in protein determination. The latter is supported by the observation that approximately 24% of total nitrogen in *C. finmarchicus* is non-protein nitrogen [26]. Consequently, the Kjeldahl method, which estimates protein content from total nitrogen using a nitrogen-to-protein conversion factor, can overestimate protein [27]. In contrast, the total amino acid (TAA)-based quantification may underestimate it because acid hydrolysis can cause deamidation of asparagine and glutamine and oxidation of tryptophan and tyrosine [28].

### 2.2. Total and Free Amino Acid Composition of Undigested Materials

Consistent with the protein content, CFH contained higher ∑TAA (502.4 mg/g DW) than FFCF (429.3 mg/g DW) and FDCF (471.6 mg/g DW) (Table 2). Glutamic acid, arginine, lysine, and alanine were consistently abundant in all materials, together accounting for approximately 41% of TAA in each. All essential amino acids (EAA) except tryptophan were detected in all materials, as tryptophan is degraded during acid hydrolysis. Overall, the amino acid profile of CFH differed from a prior study, but the levels of shared amino acids align with the batch-to-batch variability documented therein [15]. The amino acid profile of FFCF is comparable to literature values but differs in concentration [26].

In addition to TAA, the FAA composition of undigested FFCF, FDCF, and CFH was analysed (Table 3). On a DW basis, ∑FAA was highest in FFCF (208.5 mg/g DW), followed by CFH (198.9 mg/g DW) and FDCF (186.8 mg/g DW), whereas ∑EAA was highest in CFH (92.5 mg/g DW). In FFCF and FDCF, about 50% of the FAA were arginine, lysine, glycine, leucine, and alanine, whereas in CFH, about 50% were arginine, lysine, leucine, tyrosine, and alanine. Phosphoserine in FFCF and FDCF was below the detection limit (0.5%) and reported as not detected. Consistent with previous results, neither tryptophan nor cysteine was detected in FFCF, FDCF, or CFH, suggesting that both amino acids may be absent in the FAA pool of *C. finmarchicus* [15,26]. Histidine and glutamine were not detected in CFH, in line with previous findings that the FAA pool of CFH contained little or no histidine and glutamine [15]. The taurine content in CFH (7.2 mg/g DW) was slightly higher than in FFCF (6.3 mg/g DW) and FDCF (7.1 mg/g DW).

EAA (also known as indispensable amino acids) are those that humans cannot synthesize in sufficient amounts and therefore must obtain from diets. The quality of a protein can be assessed by the amino acid score, defined as the ratio of each EAA (mg per g protein) to the corresponding requirement in the reference protein proposed by FAO/WHO/UNU [20]. A score ≥ 1 indicates that the EAA meets or exceeds the reference requirement. In general, all EAA found in FFCF and FDCF scored > 1, whereas histidine in CFH scored 0.8, indicating histidine is a limiting amino acid in CFH. Since tryptophan is degraded during acid hydrolysis, its amino acid score could not be determined.

Taurine, a neutral non-proteinogenic amino acid with multiple physiological roles, is abundant in marine species such as molluscs, crustaceans, and fish [29]. The average taurine level in crustaceans, including shrimp, prawns, crabs, and lobsters, ranges from 1.4–4.0 mg/g WW [30]. The taurine levels in CFH and FFCF are below previously reported values for these materials: CFH: 7.9–11.4 mg/g DW [15]; FFCF: 11.6 mg/g DW [26]. However, FFCF, FDCF, and CFH contained more taurine than whole Antarctic krill (1.5–1.7 mg/g DW) yet substantially less than Antarctic krill hydrolysate (30.2 g/kg protein; equivalent to approximately 24.6 mg/g DW) [31,32]. Taurine is also a taste contributor, imparting mostly sourness [33].

The contribution of individual amino acids to food taste is well established, with the tastes commonly described as bitter, sweet, sour, salty, or umami. Arginine, lysine, and leucine contribute mainly to bitterness with a slight sweet note [34]. Glutamine, glutamic acid, asparagine, and aspartic acid are known to elicit a pronounced umami taste in foods, with a secondary sour taste [34]. Alanine and glycine impart pronounced sweetness and may enhance umami through synergistic effects with other amino acids [35]. The FAA profiles suggest that umami and bitter notes may be predominant in the tested materials.

### 2.3. Changes in Degree of Hydrolysis During In Vitro Gastrointestinal Digestion

Subsequently, the gastrointestinal stability of these materials was assessed using a static in vitro gastrointestinal digestion model by tracking DH and FAA over time.

**Figure 1:**
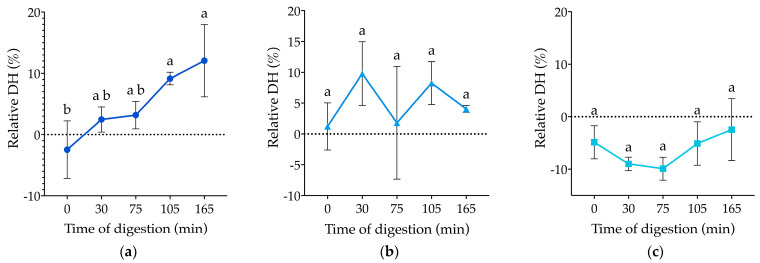
Relative degree of hydrolysis (DH, %) during in vitro gastrointestinal digestion of (**a**) fresh-frozen *C. finmarchicus*, (**b**) freeze-dried *C. finmarchicus*, and (**c**) *C. finmarchicus* hydrolysate measured at 0, 30, 75, 105, and 165 min. Results are the mean ± SD from three independent digestions (*n* = 3) and were baseline corrected by subtracting the 0 min enzyme control. Within each material, different letters indicate significant differences across time points (one-way ANOVA with Tukey’s post hoc test, *p* < 0.05).

As shown in Figure 1, DH for FFCF, FDCF, and CFH was baseline-corrected to the 0-min enzyme control and is presented as relative DH (changes from baseline). Thus, negative values indicate a decrease relative to the respective control sample. Changes in DH during digestion remained limited, particularly for CFH. The relative DH values for FFCF increased steadily, reaching 12% by the end of digestion. In contrast, the relative DH of FDCF peaked at 10%, fluctuated during digestion, and ended at 4%. Given the substantial variability among the three independent digestions at 75 min (standard deviation (SD) ≈ 9%), the apparent trend should be interpreted with caution. Notably, the relative DH of CFH remained negative throughout digestion.

During gastrointestinal digestion, dietary proteins are enzymatically hydrolyzed to smaller peptides and FAA, increasing the number of free amino groups. The relative DH profiles for FFCF and FDCF are consistent with the hypothesis that both materials release additional peptides during digestion. The negative DH observed for CFH indicates no detectable net increase in primary amino groups relative to baseline. The limited release of primary amino groups likely reflects that CFH had been hydrolyzed before digestion. Additionally, the ash fraction contains about 90% salt, and the resulting high ionic strength may suppress protease activity during digestion [14,36].

### 2.4. Free Amino Acid Composition During In Vitro Gastrointestinal Digestion

Furthermore, the FAA composition of the soluble fractions of FFCF, FDCF, and CFH was measured at the beginning, midpoint, and end of digestion (Table 4). The ∑FAA in FFCF increased significantly from 1.7 mg/mL at 0 min to 1.8 mg/mL and to 2.3 mg/mL at 165 min. On the contrary, the changes in ∑FAA in FDCF and CFH throughout digestion were not statistically significant (*p* > 0.05). The ∑FAA in CFH decreased from 7.0 mg/mL at 0 min to 6.6 mg/mL at 75 min and increased to 7.2 mg/mL at 165 min. In FDCF, ∑FAA rose slightly from 8.4 to 9.9 mg/mL at the midpoint but dropped to 9.8 mg/mL by the end. ∑EAA, as a subset of the FAA pool, exhibited a time course that mirrored the ∑FAA profile in each material.

**Figure 2:**
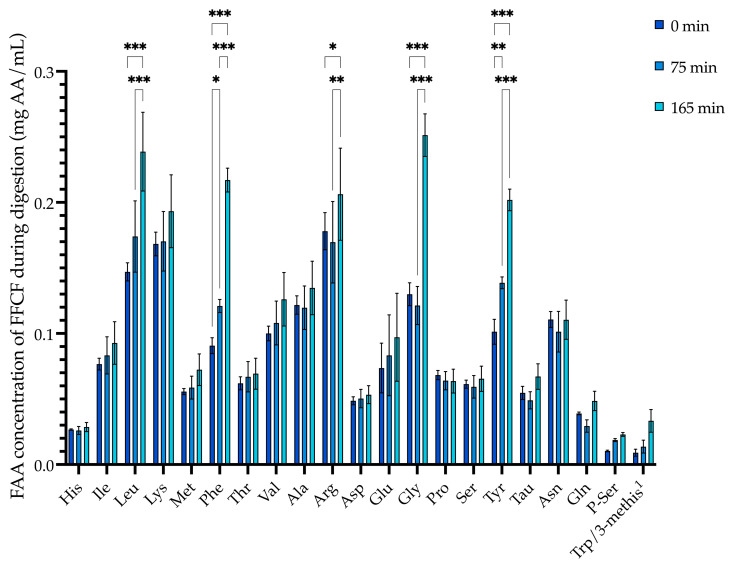
Free amino acid (FAA) composition of fresh-frozen *C. finmarchicus* (FFCF) during in vitro gastrointestinal digestion at 0, 75, and 165 min. Results are the mean ± SD from three independent digestions (*n* = 3). The FAA concentration at 165 min was corrected with a dilution factor of 1.3. Within each amino acid, asterisks denote significant differences among time points (one-way ANOVA with Tukey’s post hoc test, * *p* < 0.05, ** *p* < 0.01, and *** *p* < 0.001). ^1^ Tryptophan and 3-methyhistidine co-eluted under the chromatographic conditions.

Changes in individual FAA in FFCF during digestion are presented in Figure 2. The levels of leucine, phenylalanine, arginine, glycine, and tyrosine increased significantly.

**Figure 3:**
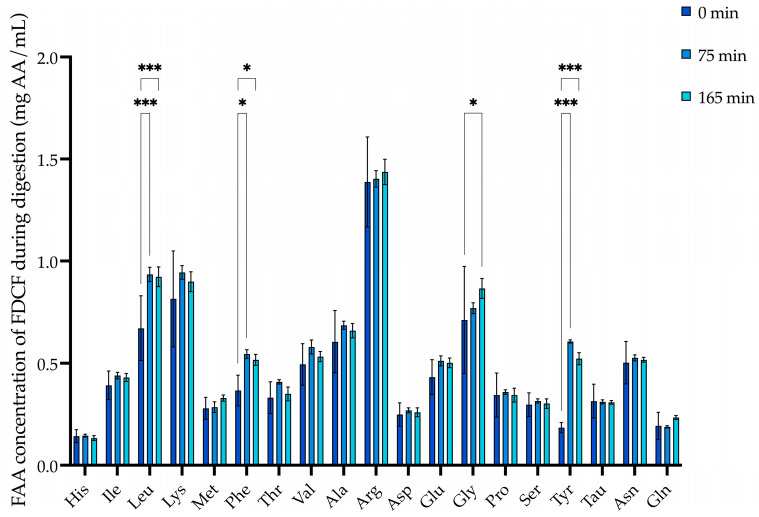
Free amino acid (FAA) composition of freeze-dried *C. finmarchicus* (FDCF) during in vitro gastrointestinal digestion at 0, 75, and 165 min. Results are the mean ± SD from three independent digestions (*n* = 3). The FAA concentration at 165 min was corrected with a dilution factor of 1.3. Within each amino acid, asterisks denote significant differences among time points (one-way ANOVA with Tukey’s post hoc test, * *p* < 0.05 and *** *p* < 0.001).

Figure 3 shows changes in individual FAA in FDCF during digestion. From the start (0 min) to the end of digestion (165 min), leucine, phenylalanine, glycine, and tyrosine increased significantly.

**Figure 4:**
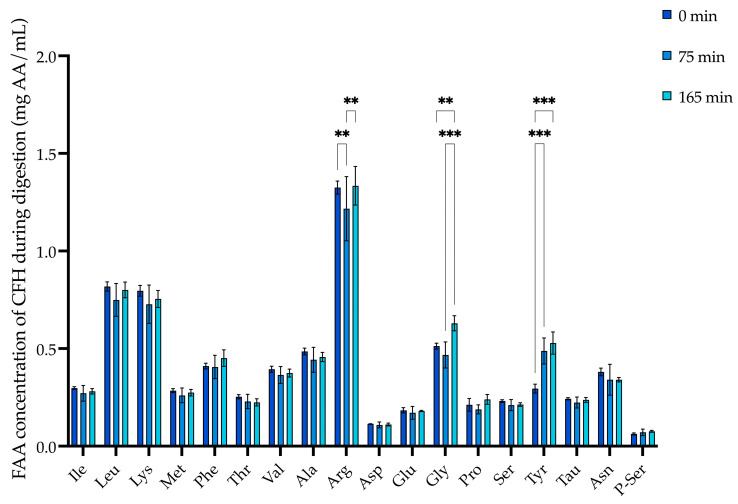
Free amino acid (FAA) composition of *C. finmarchicus* hydrolysate (CFH) during in vitro gastrointestinal digestion at 0, 75, and 165 min. Results are the mean ± SD from three independent digestions (*n* = 3). The FAA concentration at 165 min was corrected with a dilution factor of 1.3. Within each amino acid, asterisks denote significant differences among time points (one-way ANOVA with Tukey’s post hoc test, ** *p* < 0.01 and *** *p* < 0.001).

Figure 4 illustrates variations in individual FAA in CFH during digestion. From 0 min to 165 min, arginine, glycine, and tyrosine increased significantly.

Throughout digestion, arginine, lysine, and leucine were the most abundant amino acids in the soluble fractions of CFH, FFCF, and FDCF, consistent with the FAA profiles of the undigested materials. Overall, the increases in the concentrations of certain FAA at the end of digestion likely reflect their release from proteins and peptides. The digestive enzyme pepsin is inactivated at neutral pH and can be proteolytically degraded during the intestinal phase, thereby contributing to the FAA pool and complicating the attribution of FAA changes to the raw materials.

To complement the DH and FAA results, the antioxidant (FRAP, ORAC) and antidiabetic (DPP-IV, PTP1B) activities were evaluated throughout digestion.

### 2.5. Influence of In Vitro Gastrointestinal Digestion on Antioxidative Capacity

**Figure 5:**
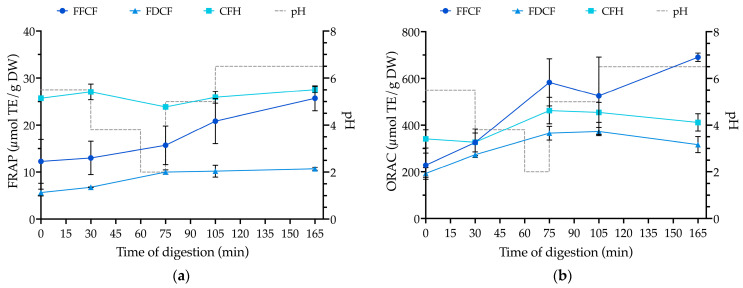
Changes in antioxidant capacity of fresh-frozen *C. finmarchicus* (FFCF), freeze-dried *C. finmarchicus* (FDCF), and *C. finmarchicus* hydrolysate (CFH) during in vitro gastrointestinal digestion, measured by (**a**) ferric reducing antioxidant power (FRAP) assay and (**b**) oxygen radical absorbance capacity (ORAC) assay. Values are the mean ± SD from three independent digestions (*n* = 3) and are expressed as µmol Trolox equivalent (TE) per g dry weight (µmol TE/g DW). Statistical analysis was performed with one-way ANOVA with Tukey’s post hoc (*p* < 0.05).

CFH had the highest initial FRAP antioxidant capacity (25.7 µmol Trolox equivalents (TE)/g DW) among all materials and remained the highest throughout digestion, ending at 27.5 µmol TE/g DW (Figure 5a). The initial FRAP value of FFCF (12.3 µmol TE/g DW) climbed to 15.7 µmol TE/g DW at 75 min and to 25.7 µmol TE/g at 165 min. In contrast, FDCF exhibited the lowest antioxidant capacity throughout digestion: its initial FRAP value (5.7 µmol TE/g DW) rose to 10.7 µmol TE/g DW by the end of digestion.

To complement the FRAP results, we assessed the antioxidant capacity of FFCF, FDCF, and CFH during digestion using the ORAC assay (Figure 5b). The ORAC antioxidant capacity of FFCF increased from 228 µmol TE/g DW at 0 min to 583 µmol TE/g DW at 75 min, surpassing CFH and FDCF, and remained highest thereafter, ending at 690 µmol TE/g DW. Given the substantial variation at 105 min (SD = 166 µmol TE/g DW, among three independent digestions), the apparent trendline should be interpreted with caution. CFH had the highest initial ORAC antioxidant capacity (341 µmol TE/g DW), showed a small drop at 30 min, peaked at 75 min (462 µmol TE/g DW), and declined to 411 µmol TE/g DW at the end of digestion. The ORAC antioxidant capacity time course for FDCF tracked that of CFH but remained the lowest throughout digestion, increasing from 193 (at 0 min) to 317 µmol TE/g DW (at 165 min).

FRAP and ORAC assays yielded different antioxidant capacity results in all materials. Such differences are expected given their distinct mechanisms. The FRAP assay quantifies the reducing power via an electron transfer (ET) mechanism at pH 3.6, whereas ORAC quantifies peroxy-radical scavenging through a hydrogen-atom transfer (HAT) mechanism at pH 7.4 [37]. After 75 min of digestion, the slight decrease in ORAC and the moderate increase in FRAP in FDCF and CFH suggest that antioxidative amino acid residues and side chains have been sufficiently exposed from proteins and peptides, highlighting a shift in the antioxidant mechanism [38].

Among the selected materials, FDCF had the lowest antioxidant capacity in both assays at all time points. Although vacuum freeze-drying is a gentle dehydration method because it removes water by low-temperature sublimation, the loss of water molecules through sublimation can alter microstructure, potentially destabilizing bioactive molecules and degrading certain antioxidant compounds [39,40]. A few studies reported that freeze-dried materials exhibit lower antioxidant capacity than their frozen counterparts [39,41]. A comparable time course has been observed in the red sea cucumber (*Parastichopus tremulus*), with freeze-dried samples displaying lower FRAP and ORAC values than their fresh-frozen counterparts throughout simulated digestion [42].

The antioxidant capacity of CFH and its native biomasses is in the range of other protein hydrolysates from marine rest raw materials, such as shrimp cephalothorax hydrolysate (FRAP: 5.4–9.8 µmol TE/g WW) [43], shrimp waste (ORAC: 16.9 µmol TE/g WW) [44], krill protein hydrolysate (ORAC: 374 µM TE/mg WW) [45], and salmon trimming hydrolysate (ORAC: 198 µmol TE/g WW) [46].

All in all, the FRAP and ORAC results confirmed that CFH retained its initial antioxidant capacity throughout digestion (*p* ≥ 0.05). In contrast, the antioxidant capacity of FFCF and FDCF was improved by the end of digestion to varying degrees (*p* < 0.05).

### 2.6. Influence of In Vitro Gastrointestinal Digestion on Antidiabetic Activity

Table 5 summarizes the DPP-IV inhibitory activity of FFCF, FDCF, and CFH during digestion, reported as IC_50_ in mg/mL. FFCF and CFH exhibited their strongest DPP-IV inhibitory activity at 75 min, with IC_50_ of 0.84 and 1.87 mg/mL, respectively. In contrast, FDCF showed its most potent DPP-IV inhibitory activity at 165 min (IC_50_ = 1.58 mg/mL). In all materials, IC_50_ values tended to decrease during digestion; however, within each material, the changes relative to the initial values were not statistically significant (*p* ≥ 0.05).

Additionally, antidiabetic activity was evaluated using the PTP1B inhibition assay with a T-cell protein tyrosine phosphatase (TC-PTP) counter-screen to assess selectivity, as preferential inhibition of PTP1B over TC-PTP is typically desirable in antidiabetic drug development to minimize immunological side effects [47,48].

At the tested assay concentration (1 mg/mL), PTP1B inhibition was observed in CFH and FFCF only at 0 and 30 min of digestion, while FDCF showed no activity at any time point tested (Tables S1–S3). Counter-screening against TC-PTP showed that all PTP1B-positive samples also inhibited TC-PTP, indicating limited selectivity. Moreover, the subsequent loss of inhibition at later time points indicates that the inhibitors are labile to gastrointestinal digestion, which lowers the likelihood that oral exposure would induce TC-PTP-mediated immunological effects. Nonetheless, these findings derive from in vitro assays and require confirmation in cell-based and in vivo models before assertive conclusions can be drawn. Overall, under our in vitro conditions, CFH and its native biomasses (FFCF, FDCF) are unlikely to exert dietary antidiabetic effects via selective inhibition of PTP1B.

On the other hand, FFCF, FDCF, and CFH exhibited DPP-IV inhibitory activity throughout digestion, with the IC_50_ values within the range reported for other protein hydrolysates, including Antarctic krill hydrolysate (1.63 mg/mL) [49], salmon protein hydrolysate (1.01 mg/mL) [50], and whey protein hydrolysate (1.28 mg/mL) [51]. Furthermore, CFH exhibited stronger DPP-IV inhibitory activity than laboratory-scale *C. finmarchicus* hydrolysates evaluated under a different in vitro digestion protocol (INFOGEST; IC_50_ = 3.44–4.27 mg/mL) [52]. In our in vitro model, the 75-min time point corresponds to digesta entering the small intestine, where small peptides and FAA are absorbed. Because DPP-IV inhibitory activity in FFCF and CFH peaked at 75 min and DPP-IV is expressed in the small intestine [53], the inhibitors appear stable during digestion and may exert systemic effects after absorption.

### 2.7. Association Between DH/FAA and Bioactivities During Digestion

To assess whether digestion indicators tracked the demonstrated bioactivity, we computed Pearson’s correlations between changes in DH and FAA over the digestion time course and antioxidant capacity (FRAP, ORAC) and DPP-IV inhibitory activity. Any significant correlations were interpreted as associative, not causal (full correlation matrices are provided in Tables S4–S6).

During digestion, DH in FFCF showed moderate, positive correlations with FRAP (r = 0.619, *p* < 0.05) and ORAC (r = 0.686, *p* < 0.05). In FDCF, DH correlated moderately and positively with ORAC (r = 0.624, *p* < 0.05) but not with FRAP. In CFH, DH was not significantly correlated with either FRAP or ORAC. No significant correlation between DH and DPP-IV inhibitory activity was observed in any material.

Next, we examined whether changes in FAA paralleled changes in bioactivity by correlating individual FAA with FRAP, ORAC, and DPP-IV throughout digestion.

In FFCF, FRAP showed strong, significant correlations with tyrosine (r = 0.903), phenylalanine (r = 0.857), and glycine (r = 0.748). Its ORAC correlated significantly with leucine (r = 0.680), phenylalanine (r = 0.792), and tyrosine (r = 0.873). In FDCF, leucine (r = 0.772), phenylalanine (r = 0.807), and tyrosine (r = 0.917) showed significant correlations with FRAP, while phenylalanine (r = 0.759) and tyrosine (r = 0.929) associated significantly with ORAC. In CFH, no amino acids displayed a significant correlation with ORAC; however, glycine was strongly associated with FRAP (r = 0.838, *p* < 0.05). For DPP-IV, only CFH showed a significant correlation with tyrosine (r = −0.865).

Overall, a higher DH correlated significantly with higher FRAP and ORAC in FFCF but only with ORAC in FDCF, supporting, but not proving, that the peptides generated during digestion positively correlated with antioxidant capacity. Given that the changes in DH and antioxidant capacity for CFH were small and non-linear (*p* > 0.05), the lack of a significant correlation is not unexpected. Moreover, DH showed no significant association with DPP-IV inhibitory activity in any of the tested materials. This is reasonable because DPP-IV inhibitory activity reflects the properties of the peptides produced, such as peptide sequence, molecular weight, and the presence of hydrophobic/aromatic residues near the N-terminus, more than DH per se [54,55].

During gastrointestinal digestion, bioactive peptides and amino acids can be released from intact proteins and exhibit bioactivity. However, bioactive peptides already present in protein hydrolysates may be further degraded during proteolysis, potentially reducing their bioactivity because peptides are more potent antioxidants than amino acids [56]. Although the antioxidant activity of individual amino acids has been measured using in vitro assays [57,58], these findings cannot be directly translated to a food matrix because of matrix complexity and synergistic interactions among components.

### 2.8. Limitations

This study has three methodological limitations relevant to the interpretation of results. First, OPA-based DH provided an estimation of newly generated primary amino groups, because OPA does not react with secondary amines (e.g., proline) but does react with N-terminal primary amines and the epsilon-amino groups of lysine side chains [59,60]. Second, the current study used a non-INFOGEST in vitro digestion protocol, which may limit comparability with INFOGEST-based studies [61]. Third, despite being simple, controllable, and reproducible in vitro gastrointestinal digestion does not replicate in vivo dynamics such as the gradual release of gastric juice, peristalsis, absorption, and gastric emptying. Consequently, the results should be interpreted within the context of in vitro gastrointestinal conditions and not directly extrapolated to in vivo settings.

### 2.9. Future Research Directions

To build on the antioxidant capacity (FRAP, ORAC) and antidiabetic (DPP-IV) activity observed under our digestion conditions, future work should: (i) desalt materials, particularly CFH, to reduce the high ash content; (ii) perform bioactivity-guided purification and peptide identification by liquid chromatography-tandem mass spectrometry (LC-MS/MS); and (iii) assess Caco-2 permeability, stability in model food matrices, cytotoxicity, and allergenicity (e.g., tropomyosin) before proceeding to in vivo studies.

## 3. Materials and Methods

### 3.1. Raw Materials

The fresh-frozen *C. finmarchicus* biomass used in this study was purchased from Zooca (Calanus AS, Tromsø, Norway). *C. finmarchicus* biomass was collected in Vestfjorden, Nordland, Norway (≤10 m depth) by slow trawling with a fine-meshed net between 19 and 22 May 2022, and was frozen on board into blocks within minutes. One frozen block (25 kg) was transported to the Arctic University of Norway (UiT, Tromsø, Norway) and stored at −20 °C until analysis. A portion of the frozen biomass was vacuum freeze-dried using a ScanVac CoolSafe™ (110-4 LaboGene™, Allerød, Denmark) to obtain freeze-dried *C. finmarchicus*. FDCF was finely ground and stored at −20 °C until further analysis. *C. finmarchicus* hydrolysate was provided by Zooca in October 2024 and stored at −37 °C until analysis.

### 3.2. Chemicals

The porcine digestive enzymes pepsin (P6887), pancreatin (P1750), and bile extract (B8631), as well as (±)-6-hydroxy-2,5,7,8-tetramethylchroman-2-carboxylic acid (Trolox, 97%), 2,2′-azobis(2-methylpropionamidine) dihydrochloride (AAPH, 98%), physiological amino acid standards (A6407 and A6282), 5-sulfosalicylic acid dihydrate (SSA), fluorescein sodium salt, potassium chloride (KCl), calcium chloride dihydrate (CaCl_2_·2H_2_O), ο-phthaldialdehyde (OPA) reagent (P7914), Gly-Pro-7-amido-4-methylcoumarin hydrobromide (Gly-Pro-AMC HBr), dipeptidyl peptidase IV (DPP-IV) human recombinant, L-serine (S4500, ≥99%), DL-norleucine (N1398, ≥98%), sodium bicarbonate (NaHCO_3_), and sodium hydroxide (NaOH) were purchased from Sigma-Aldrich (Darmstadt, Germany).

Hydrochloric acid (HCl, 37%, ACS reagent), sodium chloride (NaCl), 1,4-dithiothreitol (DTT, 97%), dichloromethane (DCM, 100.0%), and methanol (MeOH, 99.9%) were purchased from VWR Chemicals (Darmstadt, Germany). Acetic acid (99.8%) and sodium phosphate dibasic dodecahydrate (Na_2_HPO_4_·12H_2_O, ≥99.0%) were obtained from Honeywell (Seelze, Germany).

Sodium acetate (C_2_H_3_NaO_2_, anhydrous, ≥99.0%), 4-(2-hydroxyethyl)piperazine-1-ethanesulfonic acid (HEPES), bovine serum albumin (BSA), ethylenediaminetetraacetic acid (EDTA), dimethyl sulfoxide (DMSO), protein tyrosine phosphatase 1B (PTP1B, 539735), PTP inhibitor IV (540211), potassium dihydrogen phosphate (KH_2_PO_4_, ≥ 99.5%), iron (III) chloride hexahydrate (FeCl_3_·6H_2_O, ACS reagent), and 2,4,6-tris(2-pyridyl)-s-triazine (TPTZ, ≥98%) were bought from Merck KGaA (Darmstadt, Germany).

Magnesium chloride hexahydrate (MgCl_2_·6H_2_O, ≥99.0%) was supplied by Avantor Performance Materials LLC (Gliwice, Poland), di-potassium hydrogen phosphate (K_2_HPO_4_, >99.0%) by Fluka Chemie AG (Buchs, Switzerland), and UltraPure™ 6,8-difluoro-4-methylumbelliferyl phosphate (DiFMUP, D6567) by Thermo Fisher Scientific (Waltham, MA, USA). Diprotin A (Ile-Pro-Ile) was from Bachem (Bubendorf, Switzerland). Lithium loading buffer (pH 2.2) was from Biochrom (Cambridge, UK).

### 3.3. Proximate Composition Analysis

#### 3.3.1. Determination of Water and Ash Content

Briefly, 10 g of FFCF (*n* = 9), 2 g of FDCF (*n* = 5), and 2 g of CFH (*n* = 5) were dried at 105 °C until reaching constant weights, and the weight losses were calculated as water [62]. Subsequently, the water-free sample was combusted at 540 °C for 16 h in a muffle furnace, and the remaining ash was weighed [63].

#### 3.3.2. Determination of Lipid Content

The lipid extraction method developed by Folch et al. [64] was performed with modifications described by Wang et al. [19]. Approximately 0.5 g of FFCF or CFH was homogenized in 10 mL DCM:MeOH (2:1, *v*/*v*) using an Ultra Turrax^®^ (T 25 basic IKA^®^—Werke GmbH & Co. KG, Staufen, Germany) and shaken at 1373 rpm for 25 min using a Multi Reax (Heidolph, Schwabach, Germany). FDCF (70 mg) samples were rehydrated to 0.5 g using Milli-Q water before homogenizing with the solvents. Subsequently, the samples were centrifuged at 15,000× *g* at 20 °C for 10 min. The supernatant was collected and mixed with 0.9% NaCl solution (2.06 mL for FFCF and FDCF and 2.23 mL for CHF). The mixture was gently inverted four times before centrifuging at 4500× *g* at 20 °C for 10 min. Then, the lower phase containing lipids was transferred into a glass vial and evaporated under nitrogen gas. The total lipid content of each raw material was determined gravimetrically through six technical replicates (*n* = 6).

#### 3.3.3. Determination of Protein Content

Protein content was determined based on the TAA composition determined using a Biochrom 30+ amino acid analyzer (Cambridge, UK) as described before by Mæhre et al. [65].

### 3.4. Determination of Total and Free Amino Acid Composition

Acid hydrolysis was carried out according to Mæhre et al. [27], modified from Moore and Stein [66], to release all amino acids in a sample prior to TAA analysis. DL-norleucine at a concentration of 20 mM was used as an internal standard. In brief, 200 mg of FFCF or CFH was mixed with 0.5 mL of Milli-Q water, while 40 mg of FDCF was mixed with 0.7 mL of Milli-Q water in Duran^®^ glass tubes (DWK Life Science GmbH, Wertheim/Main, Germany). Then, 0.5 mL of 20 mM DL-norleucine and 1.2 mL of 37% HCl were added to each sample. The mixture was flushed with nitrogen gas for 10 to 15 s, capped, and set on a heating block at 110 °C for 24 h. After cooling down, a 1 mL aliquot of the supernatant was transferred to an Eppendorf^®^ tube (Eppendorf AG, Hamburg, Germany) and centrifuged at 14,000× *g* at 20 °C for 5 min. Subsequently, 0.1 mL of supernatant was transferred to a glass vial, evaporated under nitrogen gas, resuspended in 1 mL of lithium loading buffer (pH 2.2), and stored at 4 °C until analysis.

The FAA composition was analyzed as described by Mæhre et al. [67]. FFCF (1 g), FDCF (200 mg), and CFH (1 g) were each mixed with 1 mL of 20 mM DL-norleucine and 9 mL of Milli-Q water in a 50 mL centrifuge tube and homogenized using the Ultra Turrax for 15 s. Subsequently, 1 mL of 35% SSA was added to each sample, followed by centrifugation at 14,000× *g* at 20 °C for 5 min. A 200 µL aliquot of the supernatant was transferred to a glass vial, mixed with 800 µL of lithium loading buffer, and stored at 4 °C until analysis.

For the digestion samples, an aliquot of 180 µL was mixed with 20 µL of 20 mM DL-norleucine and 20 µL of 35% SSA in an Eppendorf tube, then the mixture was vortexed and centrifuged at 14,000× *g* at 20 °C for 5 min. A 100 µL aliquot of the supernatant was transferred to a glass vial, mixed with 100 µL of lithium loading buffer, and stored at 4 °C until analysis.

### 3.5. In Vitro Gastrointestinal Digestion

The static in vitro gastrointestinal digestion model was carried out as described by Jensen et al. [68]. The pH during digestion was adjusted using 1 M and 0.1 M HCl and NaOH solutions.

Briefly, 1 g of FFCF, FDCF, and CFH and 1 mL Milli-Q water (negative control) were each mixed with 15 mL of pepsin solution (4.62 g/L pepsin, 49 mM NaCl, 12 mM KCl, 10 mM CaCl_2_, 2.4 mM MgCl_2_, and 3.5 mM K_2_HPO_4_). All mixtures were incubated at 37 °C under constant stirring throughout the simulated digestion process. The pH of the digestion mixture was adjusted to 5.5 before collecting a 3 mL sample (0 min). The remaining mixture was incubated for 30 min. After collecting a 4 mL sample (30 min), the pH of the remaining digestion solution was adjusted to 3.8, and the mixture was incubated for another 30 min. Then, pH was adjusted to 2.0, and the mixture was incubated for 15 min before collecting a 3 mL sample (75 min). Subsequently, 1.5 mL of bile/pancreatin solution (containing 25 g/L bile extract, 4 g/L pancreatin, and 0.1 mM NaHCO_3_) was added to the digestion solution, and pH was adjusted to 5.0 before further incubation. After 30 min, a 3 mL sample was collected (105 min) and pH adjusted to 6.5. After incubating for another 60 min, the remaining solution was collected (165 min). To terminate the enzymatic digestion, all samples were heated in a water bath at 90 °C for 5 min immediately after collection and then placed on ice. The inactivated samples were centrifuged at 4500× *g* for 15 min at 4 °C, and the supernatants were stored at −37 °C until further analyses. Digestion of each raw material was performed in three independent replicates (*n* = 3).

### 3.6. OPA-Based Degree of Hydrolysis

The extent of hydrolysis of selected materials during digestion was quantified as DH using the OPA method [59,69]. The OPA assay can quantify primary amino groups by forming OPA adducts that exhibit strong absorbance at 340 nm.

All reactions took place in phosphate-buffered saline (PBS) solution, pH 7.4, composed of 10 mM Na_2_HPO_4_∙12H_2_O, 1.8 mM KH_2_PO_4_, 137 mM NaCl, and 2.7 mM KCl. L-serine was used as a standard and a positive control (0.4 to 0.025 mg/mL). Briefly, 30 µL of standard, sample, or negative control (PBS) was added to each reaction well of a 96-well plate. To minimize the edge effect, only the inner 60 wells were used. The incomplete OPA reagent was activated by adding 0.93 mg of DTT to each mL of the final OPA reagent needed. Subsequently, 225 µL of activated OPA reagent was transferred to all wells using a multichannel pipette. The reaction plate was incubated at 26 °C for 5 min inside the microplate reader (SpectraMax^®^i3 Multi-Mode Microplate Reader, Molecular Device, LLC, San Jose, CA, USA) before measuring the absorbance at 340 nm. Three technical replicates were performed for each sample from individual digestion (*n* = 3).

Subsequently, relative DH was calculated according to Benjakul and Morrissey with baseline correction [70]:

(1) $$Relative\;DH\%=\left[{(Ser-NH}_{2}^{t}-{Ser-NH}_{2}^{0})/({Ser-NH}_{2}^{tot}-{Ser-NH}_{2}^{0})\right]\times 100$$

where ${\mathrm{Ser}-\mathrm{NH}}_{2}^{t}$ is the concentration of free primary amino groups released at a certain time t; ${\mathrm{Ser}-\mathrm{NH}}_{2}^{0}$ is the concentration of free primary amino groups at time 0 (the control group comprised a heat-inactivated pepsin solution and approximately 1 g of relevant raw material); ${\mathrm{Ser}-\mathrm{NH}}_{2}^{tot}$ is the concentration of total primary amino groups after complete acid hydrolysis as described in 3.4.

### 3.7. Screening for Antioxidant Capacity

#### 3.7.1. Ferric Reducing Antioxidant Power Assay

The FRAP assay was carried out following the modified protocol described by Jensen et al. [68]. Trolox (15.625 to 1000 µM) was used as a standard (positive control) and Milli-Q water as a negative control. The FRAP reagent was prepared by mixing 20 mM FeCl_3_·6H_2_O solution, 10 mM TPTZ solution, and 300 mM acetate buffer (pH 3.6) in a ratio of 1:1:10, and it was kept at 37 °C for at least 10 min before use. The reaction took place in a clear 96-well microplate, where 5 µL of the digestion sample, Milli-Q water, or Trolox standard was mixed with 15 µL of Milli-Q water and 150 µL of FRAP reagent. The plate was incubated at 37 °C for 30 min in a dark environment before being read at 595 nm using the microplate reader. The average antioxidant capacity of three independent replicates (*n* = 3) was normalized to DW and expressed as average µmol TE per gram raw material.

#### 3.7.2. Oxygen Radical Absorbance Capacity Assay

The ORAC assay, originally elucidated by Dávalos et al. [71], was applied in this study with modifications described by Jensen et al. [68]. The 75 mM phosphate buffer (pH 7.4) was used as a solvent, and Trolox (6.25 to 100 µM) was used as a standard (positive control). The reaction took place in a black 96-well flat-bottom microplate (Nunc^®^MaxiSorp™, Thermo Fisher Scientific, Waltham, MA, USA), where 25 µL of the digestion sample or Trolox standard was mixed with 125 µL fluorescein sodium salt solution (final assay concentration 55 nM), and 200 µL of phosphate buffer was added as a negative control. The plate was incubated at 37 °C for 15 min in the dark. Subsequently, 50 µL of AAPH reagent (final assay concentration 38 nM) was added to all wells using a multichannel pipette. The fluorescence was measured in the microplate reader with kinetic reading at 485 and 520 nm every 60 s for 80 min. Only the inner 60 wells were used to avoid edge effects. The antioxidant capacity was expressed as µmol TE per gram of DW raw material, with an average of three independent replicates (*n* = 3).

### 3.8. Screening for Antidiabetic Activity

The supernatant samples collected during digestion were freeze-dried, resuspended in Milli-Q water to a concentration of 10 mg/mL, and stored at −20 °C before analyses.

#### 3.8.1. Dipeptidyl Peptidase-IV Inhibitory Assay

DPP-IV is a serine protease that inactivates incretin hormones, including glucose-independent insulinotropic polypeptide (GIP) and glucagon-like peptide-1 (GLP-1), by removing N-terminal dipeptides [72]. Inhibiting DPP-IV preserves incretin activity and supports glycemic regulation.

The DPP-IV inhibitory activity assay was carried out as described by Harnedy and FitzGerald [73] with minor modifications. Diprotin A (final assay concentration 5 µM) was used as a positive control. Briefly, the reaction was conducted in a black 96-well microplate, where 10 µL of the digestion sample or Diprotin A was mixed with 30 µL of 0.02 M Tris-HCl assay buffer (pH 8.0, containing 0.10 M NaCl and 1 mM EDTA), and 50 µL of 200 µM Gly-Pro-AMC HBr (G-P-AMC). The full DPP-IV activity was determined by a max reaction control group, containing 40 µL of assay buffer and 50 µL of G-P-AMC. The background group contained 50 µL of assay buffer and 50 µL of G-P-AMC. The plate was incubated at 37 °C for 5 min. The fluorescence was recorded before (T0) adding 10 µL of DPP-IV (0.8 unit/µL) and after a 30-min incubation (T30) with DPP-IV, using a Victor^®^ microplate reader (PerkinElmer, Singapore) with excitation and emission wavelengths set at 355 nm and 460 nm, respectively. The results, derived from three individual digestion replicates (*n* = 3), were expressed as IC_50_ in mg/mL. IC_50_ was calculated based on the maximum reaction control computed from their fluorescent readouts using regression.

#### 3.8.2. Protein Tyrosine Phosphatase 1B Inhibitor Assay

PTP1B can dephosphorylate insulin receptors and their downstream substrates, thereby negatively regulating insulin signaling. Consequently, inhibiting PTP1B may enhance insulin sensitivity and improve glucose homeostasis [48].

The PTP1B inhibitor assay was carried out according to the protocol of Minor [74] and repeated three times for each sample. All reactions took place in PTP1B assay buffer (pH 7.2), containing 25 mM HEPES, 50 mM NaCl, 2 mM DTT, 2.5 mM EDTA, and 0.01 mg/mL BSA. The assay buffer was used as a negative control, and the PTP1B enzyme was used as a positive control. In a reaction well of a black 96-well microplate, 25 µL of sample solution (final assay concentration 1 mg/mL), 25 µL of PTP inhibitor IV solution (final assay concentration 0.41 µg/mL), or 25 µL of assay buffer was mixed with 50 µL of PTP1B enzyme solution (final assay concentration 15 ng/mL). The plate was incubated at 37 °C for 30 min in a dark environment. Subsequently, 25 µL of DiFMUP solution (final assay concentration 10 µM) was added to all wells, and the plate was incubated at 37 °C for 10 min. The reaction plate was read using a Tecan plate reader (Männedorf, Switzerland), where the excitation and emission wavelengths were set at 360 nm and 465 nm, respectively. The inhibitory activity of samples on the PTP1B enzyme was normalized following this formula:

(2) $$\mathrm{Activity}\;(\%)=\frac{\left({\mathrm{Average}}_{samples}-{\mathrm{Average}}_{inhibitor}\right)}{\left({\mathrm{Average}}_{negative\;control}-{\mathrm{Average}}_{inhibitor}\right)}\times 100,$$

Samples with less than 30% of the original activity were considered to have active PTP1B inhibition, activity levels between 30 and 40% were considered questionable, and activity levels above 40% were considered to have no active PTP1B inhibitors. Samples containing active PTP1B inhibitors were subsequently screened for selectivity against TC-PTP using the same procedure, with TC-PTP enzyme as a positive control.

### 3.9. Statistical Analysis

Experimental data are reported as arithmetic means ± SD on a WW or DW basis, as specified. For TAA, FAA, DH, FRAP, ORAC, and DPP-IV, one-way ANOVA was conducted within each material to compare digestion time points, with Tukey’s post hoc test for multiple comparisons. Normality was assessed using the Shapiro–Wilk test. Analyses were performed in GraphPad Prism for macOS, Version 11.0.1 (GraphPad Software, San Diego, CA, USA). Pearson correlation coefficients were computed in IBM SPSS Statistics, Version 29.0.2.0 (IBM, Armonk, NY, USA). A significance level of *p* < 0.05 was used for all statistical analyses conducted in this study.

## 4. Conclusions

This study used a static in vitro gastrointestinal digestion model to evaluate the gastrointestinal stability of CFH and the impact of digestion on antioxidant capacity (FRAP, ORAC) and antidiabetic activity (DPP-IV, PTP1B).

CFH contained significantly more protein than the native biomasses (FFCF, FDCF), and its FAA pool was enriched in EAA and taurine, highlighting its nutritional value. Nevertheless, its high ash content may hinder application in food and compromise functional properties.

Across all digestion time points, arginine, lysine, and leucine were the most abundant FAA in the soluble fraction of each material. OPA-based DH patterns were consistent with our hypothesis: CFH showed essentially no additional hydrolysis over the digestion time course, whereas FFCF and FDCF exhibited further proteolysis relative to baseline (0 min). In CFH, both FRAP and ORAC antioxidant activities were maintained through the end of digestion, and DPP-IV inhibitory activity was moderately enhanced (IC_50_ decreased from 3.79 ± 0.69 to 1.96 ± 0.28 mg/mL, *p* ≥ 0.05). PTP1B inhibition in CFH was detected only at 0 and 30 min and lacked selectivity over TC-PTP, making a dietary PTP1B-mediated mechanism unlikely. Together, these findings support CFH as a potential value-added food ingredient or nutraceutical with antioxidant and DPP-IV inhibitory activities; however, desalting, bioactivity-guided peptide identification, permeability, toxicity, allergenicity, and in vivo validation are warranted [75].

## Figures and Tables

**Table 2:** Total amino acid (TAA) composition of fresh-frozen *C. finmarchicus* (FFCF), freeze-dried *C. finmarchicus* (FDCF), and *C. finmarchicus* hydrolysate (CFH). Results are the mean ± SD (*n* = 3). Different superscript letters within a row indicate significant differences among DW-based values (one-way ANOVA with Tukey’s post hoc test, *p* < 0.05).

Amino Acids	FFCF		FDCF		CFH	
	mg/g WW	mg/g DW	mg/g WW	mg/g DW	mg/g WW	mg/g DW
Histidine	1.2 ± 0.2	9.4 ± 1.4 ^a^	8.9 ± 0.6	10.1 ± 0.7 ^a^	2.5 ± 0.0	5.3 ± 0.1 ^b^
Isoleucine	2.3 ± 0.1	18.1 ± 0.9 ^b^	16.5 ± 1.1	18.7 ± 1.3 ^b^	10.3 ± 0.2	22.3 ± 0.4 ^a^
Leucine	4.1 ± 0.3	31.9 ± 2.1 ^b^	31.0 ± 1.3	35.3 ± 1.4 ^b^	18.7 ± 0.4	40.3 ± 0.8 ^a^
Lysine	4.6 ± 0.5	36.3 ± 3.7 ^b^	35.1 ± 2.2	39.8 ± 2.5 ^ab^	20.6 ± 0.5	44.5 ± 1.2 ^a^
Methionine	1.6 ± 0.2	12.6 ± 1.4 ^b^	12.2 ± 0.5	13.9 ± 0.5 ^ab^	7.2 ± 0.4	15.6 ± 0.8 ^a^
Phenylalanine	2.2 ± 0.3	17.4 ± 2.1 ^b^	16.3 ± 1.1	18.5 ± 1.3 ^ab^	9.9 ± 0.2	21.3 ± 0.5 ^a^
Threonine	2.5 ± 0.2	19.2 ± 1.9 ^b^	18.8 ± 0.8	21.4 ± 0.9 ^ab^	11.1 ± 0.4	24.1 ± 0.8 ^a^
Valine	2.7 ± 0.3	21.5 ± 2.2 ^b^	20.7 ± 0.9	23.5 ± 1.0 ^b^	13.1 ± 0.4	28.2 ± 0.8 ^a^
Cysteine	0.7 ± 0.0	5.5 ± 0.3 ^a^	5.2 ± 0.6	5.9 ± 0.7 ^a^	1.6 ± 0.2	3.4 ± 0.4 ^b^
Alanine	4.6 ± 0.4	36.4 ± 2.9 ^a^	36.1 ± 1.7	41.0 ± 1.9 ^a^	17.3 ± 0.8	37.4 ± 1.7 ^a^
Arginine	5.6 ± 0.3	43.9 ± 2.3 ^a^	39.4 ± 3.8	44.8 ± 4.3 ^a^	21.8 ± 4.6	47.0 ± 10.0 ^a^
Aspartic acid ^1^	3.5 ± 0.3	27.6 ± 2.6 ^b^	27.0 ± 1.2	30.7 ± 1.4 ^ab^	15.7 ± 0.5	33.9 ± 1.1 ^a^
Glutamic acid ^1^	7.6 ± 0.6	59.9 ± 4.4 ^b^	58.3 ± 2.2	66.3 ± 2.5 ^b^	35.1 ± 1.6	75.8 ± 3.5 ^a^
Glycine	4.2 ± 0.4	33.0 ± 2.9 ^b^	34.4 ± 1.4	39.1 ± 1.5 ^a^	16.8 ± 0.6	36.2 ± 1.3 ^ab^
Proline	2.5 ± 0.2	19.5 ± 1.7 ^a^	18.1 ± 1.3	20.6 ± 1.4 ^a^	10.3 ± 0.2	22.3 ± 0.3 ^a^
Serine	2.3 ± 0.3	18.2 ± 2.2 ^a^	17.6 ± 1.0	20.1 ± 1.1 ^a^	10.1 ± 0.2	21.8 ± 0.5 ^a^
Tyrosine	2.5 ± 0.2	19.3 ± 1.2 ^a^	19.4 ± 0.2	22.0 ± 0.3 ^b^	10.6 ± 0.2	22.8 ± 0.3 ^a^
Taurine	1.0 ± 0.1	7.5 ± 1.3 ^b^	7.2 ± 0.7	8.1 ± 0.8 ^ab^	4.5 ± 0.1	9.7 ± 0.2 ^a^
∑EAA	21.2 ± 2.0	166.6 ± 15.4 ^b^	159.4 ± 7.9	181.1 ± 8.9 ^ab^	93.4 ± 2.2	201.7 ± 4.7 ^a^
∑NEAA	32.8 ± 2.0	257.7 ± 15.6 ^b^	250.4 ± 8.2	284.5 ± 9.3 ^ab^	137.6 ± 8.0	297.3 ± 17.2 ^a^
∑TAA ^2^	54.7 ± 3.9	429.3 ± 30.7 ^b^	415.0 ± 14.9	471.6 ± 16.9 ^ab^	232.5 ± 9.8	502.4 ± 21.1 ^a^

^1^ Asparagine and glutamine were deamidated to aspartic acid and glutamic acid during acid hydrolysis and were quantified accordingly. ^2^ Sum of proteinogenic amino acids.

**Table 3:** Free amino acid (FAA) composition of fresh-frozen *C. finmarchicus* (FFCF), freeze-dried *C. finmarchicus* (FDCF), and *C. finmarchicus* hydrolysate (CFH). Results are the mean ± SD (*n* = 3). Different superscript letters within a row indicate significant differences among DW-based values (one-way ANOVA with Tukey’s post hoc test, *p* < 0.05).

Amino Acids	FFCF		FDCF		CFH	
	mg/g WW	mg/g DW	mg/g WW	mg/g DW	mg/g WW	mg/g DW
Histidine	0.5 ± 0.0	3.6 ± 0.1 ^a^	2.8 ± 0.2	3.1 ± 0.2 ^b^	n.d.	n.d.
Isoleucine	1.2 ± 0.1	9.5 ± 0.7 ^a^	7.0 ± 0.2	7.9 ± 0.2 ^b^	4.1 ± 0.1	8.9 ± 0.1 ^ab^
Leucine	2.2 ± 0.2	16.9 ± 1.2 ^b^	12.3 ± 0.1	13.9 ± 0.1 ^c^	9.9 ± 0.2	21.5 ± 0.5 ^a^
Lysine	2.9 ± 0.1	22.6 ± 1.1 ^a^	16.2 ± 1.0	18.4 ± 1.1 ^b^	10.3 ± 0.2	22.1 ± 0.6 ^a^
Methionine	0.9 ± 0.0	7.0 ± 0.3 ^b^	5.4 ± 0.1	6.2 ± 0.1 ^c^	4.0 ± 0.0	8.7 ± 0.0 ^a^
Phenylalanine	1.1 ± 0.1	8.5 ± 0.4 ^b^	6.6 ± 0.2	7.5 ± 0.2 ^c^	5.8 ± 0.1	12.5 ± 0.2 ^a^
Threonine	1.0 ± 0.1	8.0 ± 0.5 ^a^	6.2 ± 0.3	7.0 ± 0.4 ^b^	3.3 ± 0.0	7.1 ± 0.1 ^ab^
Valine	1.5 ± 0.1	12.1 ± 0.6 ^a^	9.2 ± 0.3	10.4 ± 0.4 ^b^	5.5 ± 0.0	11.8 ± 0.1 ^a^
Alanine	2.0 ± 0.1	15.7 ± 0.4 ^a^	12.3 ± 0.6	13.8 ± 0.6 ^b^	6.4 ± 0.1	13.8 ± 0.2 ^b^
Arginine	3.6 ± 0.8	27.9 ± 6.1 ^a^	24.6 ± 7.2	27.9 ± 8.1 ^a^	14.7 ± 2.6	32.0 ± 6.0 ^a^
Aspartic acid	0.9 ± 0.1	6.7 ± 0.4 ^a^	4.8 ± 0.2	5.5 ± 0.3 ^b^	1.6 ± 0.0	3.4 ± 0.1 ^c^
Glutamic acid	1.3 ± 0.1	10.0 ± 0.7 ^a^	7.7 ± 0.6	8.8 ± 0.7 ^a^	2.6 ± 0.1	5.6 ± 0.2 ^b^
Glycine	2.1 ± 0.1	16.1 ± 0.5 ^b^	16.3 ± 0.9	18.5 ± 1.0 ^a^	6.3 ± 0.4	13.6 ± 0.8 ^c^
Proline	1.1 ± 0.2	9.0 ± 1.4 ^a^	6.9 ± 0.4	7.8 ± 0.4 ^a^	2.3 ± 0.1	5.1 ± 0.3 ^b^
Serine	1.0 ± 0.1	7.6 ± 0.7 ^a^	5.6 ± 0.3	6.4 ± 0.3 ^b^	3.1 ± 0.1	6.6 ± 0.2 ^ab^
Tyrosine	1.2 ± 0.1	9.4 ± 0.4 ^b^	7.3 ± 0.5	8.3 ± 0.6 ^b^	7.3 ± 0.1	15.8 ± 0.3 ^a^
Asparagine	1.7 ± 0.1	12.9 ± 0.9 ^a^	9.7 ± 0.9	11.0 ± 1.0 ^ab^	4.9 ± 0.1	10.7 ± 0.2 ^b^
Glutamine	0.7 ± 0.1	5.1 ± 0.8 ^a^	3.8 ± 0.9	4.3 ± 1.1 ^a^	n.d.	n.d.
Taurine	0.8 ± 0.1	6.3 ± 0.4 ^b^	6.2 ± 0.2	7.1 ± 0.3 ^a^	3.3 ± 0.2	7.2 ± 0.3 ^a^
Ammonia	0.1 ± 0.0	0.7 ± 0.1 ^b^	0.6 ± 0.1	0.6 ± 0.1 ^a^	0.5 ± 0.0	1.0 ± 0.0 ^b^
Phosphoserine	n.d.	n.d.	n.d.	n.d.	0.8 ± 0.1	1.6 ± 0.1
∑EAA	11.2 ± 0.6	88.1 ± 4.5 ^a^	65.6 ± 1.8	74.5 ± 2.0 ^b^	42.8 ± 0.1	92.5 ± 0.2 ^a^
∑NEAA	14.5 ± 0.7	120.4 ± 7.4 ^a^	98.8 ± 8.1	112.3 ± 9.2 ^a^	49.3 ± 3.6	106.4 ± 7.7 ^a^
∑FAA ^1^	26.5 ± 1.0	208.5 ± 8.0 ^a^	164.4 ± 7.8	186.8 ± 8.8 ^b^	92.1 ± 3.7	198.9 ± 7.9 ^ab^

^1^ Sum of proteinogenic amino acids. n.d. = not detected.

**Table 4:** Total free amino acids (∑FAA) and total essential amino acids (∑EAA) in fresh-frozen *C. finmarchicus* (FFCF), freeze-dried *C. finmarchicus* (FDCF), and *C. finmarchicus* hydrolysate (CFH) were measured during in vitro gastrointestinal digestion at 0, 75, and 165 min. Results are the mean ± SD from three independent digestions (*n* = 3). Statistical analyses for ∑FAA and ∑EAA were performed separately. Within each row, means with different superscript letters differ significantly (one-way ANOVA with Tukey’s post hoc test, *p* < 0.05).

	∑FAA ^1^ (mg/mL)			∑EAA (mg/mL)		
	0 Min	75 Min	165 Min	0 Min	75 Min	165 Min
FFCF	1.7 ± 0.1 ^b^	1.8 ± 0.2 ^ab^	2.3 ± 0.3 ^a^	0.7 ± 0.0 ^b^	0.8 ± 0.1 ^ab^	1.0 ± 0.1 ^a^
FDCF	8.4 ± 1.8 ^a^	9.9 ± 0.2 ^a^	9.8 ± 0.5 ^a^	3.5 ± 0.8 ^a^	4.3 ± 0.1 ^a^	4.1 ± 0.2 ^a^
CFH	7.0 ± 0.2 ^a^	6.6 ± 0.9 ^a^	7.2 ± 0.4 ^a^	3.3 ± 0.1 ^a^	3.0 ± 0.3 ^a^	3.2 ± 0.2 ^a^

^1^ Sum of proteinogenic amino acids.

**Table 5:** Dipeptidyl peptidase-IV (DPP-IV) inhibitory activity (IC_50_, mg/mL) of fresh-frozen *C. finmarchicus* (FFCF), freeze-dried *C. finmarchicus* (FDCF), and *C. finmarchicus* hydrolysate (CFH) during in vitro gastrointestinal digestion. Results are the mean ± SD from three independent digestions (*n* = 3). No statistically significant differences were detected across time points within each material (one-way ANOVA with Tukey Post Hoc test, *p* ≥ 0.05).

Dipeptidyl Peptidase IV (DPP-IV) Inhibition	IC_50_ (mg/mL)		
Time of Digestion (Min)	FFCF	FDCF	CFH
0	2.06 ± 0.97	2.51 ± 1.11	3.73 ± 0.69
30	2.03 ± 0.40	1.81 ± 0.60	2.91 ± 1.12
75	0.84 ± 0.21	1.66 ± 0.65	1.87 ± 0.28
105	1.45 ± 0.53	1.87 ± 0.97	2.47 ± 0.71
165	1.28 ± 0.34	1.58 ± 0.79	1.96 ± 0.28

## Data Availability

The data presented in this study are openly available in DataverseNO at https://doi.org/10.18710/UUEOMJ (accessed on 2 July 2026).

## Supplementary Materials

The following supporting information can be downloaded at: https://www.mdpi.com/article/10.3390/md24070240/s1, Table S1: Protein tyrosine phosphatase 1B (PTP1B) inhibitory activity and T-cell protein tyrosine phosphatase (TC-PTP) counter-screen for *C. finmarchicus* hydrolysate (CFH) during in vitro gastrointestinal digestion at 0, 30, 75, 105, and 165 min. Each time point includes three independent digestions (*n* = 3); Table S2: Protein tyrosine phosphatase 1B (PTP1B) inhibitory activity and T-cell protein tyrosine phosphatase (TC-PTP) counter-screen for fresh-frozen *C. finmarchicus* (FFCF) during in vitro gastrointestinal digestion at 0, 30, 75, 105, and 165 min. Each time point includes three independent digestions (*n* = 3); Table S3: Protein tyrosine phosphatase 1B (PTP1B) inhibitory activity and T-cell protein tyrosine phosphatase (TC-PTP) counter-screen for freeze-dried *C. finmarchicus* (FDCF) during in vitro gastrointestinal digestion at 0, 30, 75, 105, and 165 min. Each time point includes three independent digestions (*n* = 3); Table S4: Pearson correlation matrices (r) for fresh-frozen *C. finmarchicus* (FFCF) across FRAP, ORAC, DPP-IV, DH, and individual FAA; Table S5: Pearson correlation matrices (r) for freeze-dried *C. finmarchicus* (FDCF) across FRAP, ORAC, DPP-IV, DH, and individual FAA; Table S6: Pearson correlation matrices (r) for *C. finmarchicus* hydrolysate (CFH) across FRAP, ORAC, DPP-IV, DH, and individual FAA.

## Abbreviations

The following abbreviations are used in this manuscript:

Caco-2	Human epithelial colorectal adenocarcinoma cell line
CFH	*Calanus finmarchicus* hydrolysate
DH	Degree of hydrolysis
DPP-IV	Dipeptidyl peptidase-IV
DW	Dry weight
EAA	Essential amino acid
ET	Electron transfer
FAA	Free amino acid
FDCF	Freeze-dried *C. finmarchicus*
FFCF	Fresh-frozen *C. finmarchicus*
FRAP	Ferric reducing antioxidant power
GIP	Glucose-independent insulinotropic polypeptide
GLP-1	Glucagon-like peptide-1
HAT	Hydrogen-atom transfer
IC_50_	Half-maximal inhibitory concentration
LC n-3 PUFAs	Long-chain omega-3 polyunsaturated fatty acids
LC-MS/MS	Liquid chromatography-tandem mass spectrometry
NEAA	Non-essential amino acid
OPA	O-phthaldialdehyde
ORAC	Oxygen radical absorbance capacity
PBS	Phosphate-buffered saline
PTP1B	Protein tyrosine phosphatase 1B
ROS	Reactive oxygen species
SD	Standard deviation
T2D	Type-2 diabetes
TAA	Total amino acid
TC-PTP	T-cell protein tyrosine phosphatase
TE	Trolox equivalent
WW	Wet weight
