# Supplementary material for: In Vitro Gastrointestinal Digestion of Calanus finmarchicus Products: Amino Acid Composition, Degree of Hydrolysis, Antioxidant Capacity, and Antidiabetic Activity
Source: Mar Drugs. 2026 Jul 7;24(7):240. doi: 10.3390/md24070240 (PMC13412531; doi:10.3390/md24070240)
Supplement: Supplementary file 1 [file marinedrugs-24-00240-s001.zip › Table_S1_CFH_PTP1B-and-TC-PTP-Inhibitory-Activity.pdf]

Table S1. Protein tyrosine phosphatase 1B (PTP1B) inhibitory activity and T-cell protein tyrosine phosphatase (TC-PTP) counter-screen for *C. finmarchicus* hydrolysate (CFH) during *in vitro* gastrointestinal digestion at 0, 30, 75, 105, and 165 min. Each time point includes three independent digestions (*n* = 3).

Sample	Digestion time (min)	PTP1B inhibitory activity (%)	PTP1B status[1]	TC-PTP inhibitory activity (%)[2]	TC-PTP status[1]
CFH	0	3	Active	13	Active
CFH	0	-5	Active	13	Active
CFH	0	4	Active	11	Active
CFH	30	3	Active	-3	Active
CFH	30	-6	Active	13	Active
CFH	30	12	Active	15	Active
CFH	75	88	Inactive	n.m.	n.m.
CFH	75	92	Inactive	n.m.	n.m.
CFH	75	118	Inactive	n.m.	n.m.
CFH	105	85	Inactive	n.m.	n.m.
CFH	105	85	Inactive	n.m.	n.m.
CFH	105	108	Inactive	n.m.	n.m.
CFH	165	16	Active	41	Questionable
CFH	165	39	Questionable	n.m.	n.m.
CFH	165	83	Inactive	n.m.	n.m.

[1]Status thresholds applied to activity: Active < 30%; Questionable 30-40%; Inactive > 40%. Negative or > 100% can occur due to baseline correction. [2]TC-PTP was measured only for replicates classified as Active for PTP1B. n.m. = not measured.
