# Supplementary material for: In Vitro Gastrointestinal Digestion of Calanus finmarchicus Products: Amino Acid Composition, Degree of Hydrolysis, Antioxidant Capacity, and Antidiabetic Activity
Source: Mar Drugs. 2026 Jul 7;24(7):240. doi: 10.3390/md24070240 (PMC13412531; doi:10.3390/md24070240)
Supplement: Supplementary file 1 [file marinedrugs-24-00240-s001.zip › Table_S6_CFH_Pearson-Correlation-Coefficients.pdf]

Table S6. Pearson correlation matrices (r) for *C. finmarchicus* hydrolysate (CFH) across FRAP, ORAC, DPP-IV, DH, and individual FAA.

Descriptive Statistics

	Mean	Std. Deviation	N
FRAP	26.0152	1.57086	15
ORAC	404.3733	62.22486	15
DPP_IV	2.5880	.91823	15
DH	-6.2624	4.23898	15
His	.	.	0
Ile	.28341	.024455	9
Leu	.78923	.057289	9
Lys	.75937	.062953	9
Met	.27310	.023904	9
Phe	.42262	.043118	9
Thr	.23560	.025180	9
Val	.37783	.028364	9
Ala	.46158	.039895	9
Arg	1.29197	.112680	9
Asp	.11146	.008709	9
Glu	.17831	.018790	9
Gly	.53666	.082342	9

Pro	.21332	.032289	9
Ser	.21829	.018135	9
Tyr	.43711	.117366	9
Asn	.35364	.045535	9
Gln	.	.	0

Correlations

		FRAP	ORAC	DPP_IV	DH	His	Ile	Leu	Lys	Met	Phe	Thr	Val	Ala	Arg	Asp	Glu	Gly	Pro	Ser	Tyr	Asn	Gln
FRAP	Pearson Correlation	1	-.443	-.103	.420	.a	.163	.357	.191	.248	.473	-.065	.167	.144	.467	.153	.129	.838**	.640	.069	.184	-.088	.a
	Sig. (2-tailed)		.098	.715	.119	.	.676	.346	.622	.520	.199	.869	.667	.711	.205	.695	.742	.005	.063	.860	.636	.821	.
	N	15	15	15	15	0	9	9	9	9	9	9	9	9	9	9	9	9	9	9	9	9	0
ORAC	Pearson Correlation	-.443	1	-.366	-.169	.a	-.208	-.288	-.284	-.196	.108	-.226	-.259	-.253	-.272	-.152	-.082	-.078	-.155	-.283	.569	-.320	.a
	Sig. (2-tailed)	.098		.180	.547	.	.592	.453	.458	.614	.781	.559	.501	.511	.478	.695	.833	.841	.690	.460	.110	.401	.
	N	15	15	15	15	0	9	9	9	9	9	9	9	9	9	9	9	9	9	9	9	9	0
DPP_IV	Pearson Correlation	-.103	-.366	1	-.018	.a	.470	.372	.441	.389	-.178	.546	.413	.456	.259	.237	.283	-.152	-.023	.538	-.865**	.421	.a
	Sig. (2-tailed)	.715	.180		.948	.	.202	.324	.235	.301	.646	.128	.270	.217	.501	.540	.461	.695	.952	.135	.003	.260	.
	N	15	15	15	15	0	9	9	9	9	9	9	9	9	9	9	9	9	9	9	9	9	0
DH	Pearson Correlation	.420	-.169	-.018	1	.a	.514	.620	.539	.583	.678*	.398	.494	.496	.805**	.582	.374	.769*	.308	.420	.158	.234	.a
	Sig. (2-tailed)	.119	.547	.948		.	.157	.075	.134	.099	.045	.289	.177	.174	.009	.100	.321	.016	.421	.260	.684	.545	.
	N	15	15	15	15	0	9	9	9	9	9	9	9	9	9	9	9	9	9	9	9	9	0
His	Pearson Correlation	.a	.a	.a	.a	.a	.a	.a	.a	.a	.a	.a	.a	.a	.a	.a	.a	.a	.a	.a	.a	.a	.a
	Sig. (2-tailed)	.	.	.	.		.	.	.	.	.	.	.	.	.	.	.	.	.	.	.	.	.
	N	0	0	0	0	0	0	0	0	0	0	0	0	0	0	0	0	0	0	0	0	0	0
Ile	Pearson Correlation	.163	-.208	.470	.514	.a	1	.962**	.982**	.987**	.708*	.952**	.967**	.990**	.879**	.928**	.891**	.463	.359	.979**	-.090	.797*	.a
	Sig. (2-tailed)	.676	.592	.202	.157	.		<.001	<.001	<.001	.033	<.001	<.001	<.001	.002	<.001	.001	.210	.343	<.001	.817	.010	.

	N	9	9	9	9	0	9	9	9	9	9	9	9	9	9	9	9	9	9	9	9	9	0
Leu	Pearson Correlation	.357	-.288	.372	.620	.[a]	.962**	1	.958**	.980**	.798**	.868**	.942**	.957**	.917**	.884**	.887**	.638	.493	.918**	-.011	.758*	.[a]
	Sig. (2-tailed)	.346	.453	.324	.075	.	<.001		<.001	<.001	.010	.002	<.001	<.001	<.001	.002	.001	.065	.178	<.001	.977	.018	.
	N	9	9	9	9	0	9	9	9	9	9	9	9	9	9	9	9	9	9	9	9	9	0
Lys	Pearson Correlation	.191	-.284	.441	.539	.[a]	.982**	.958**	1	.986**	.715*	.951**	.994**	.997**	.872**	.926**	.888**	.476	.430	.982**	-.072	.792*	.[a]
	Sig. (2-tailed)	.622	.458	.235	.134	.	<.001	<.001		<.001	.030	<.001	<.001	<.001	.002	<.001	.001	.196	.248	<.001	.855	.011	.
	N	9	9	9	9	0	9	9	9	9	9	9	9	9	9	9	9	9	9	9	9	9	0
Met	Pearson Correlation	.248	-.196	.389	.583	.[a]	.987**	.980**	.986**	1	.793*	.928**	.978**	.985**	.900**	.924**	.888**	.558	.436	.958**	.002	.746*	.[a]
	Sig. (2-tailed)	.520	.614	.301	.099	.	<.001	<.001	<.001		.011	<.001	<.001	<.001	<.001	<.001	.001	.118	.241	<.001	.996	.021	.
	N	9	9	9	9	0	9	9	9	9	9	9	9	9	9	9	9	9	9	9	9	9	0
Phe	Pearson Correlation	.473	.108	-.178	.678*	.[a]	.708*	.798**	.715*	.793*	1	.558	.715*	.696*	.829**	.770*	.690*	.837**	.520	.601	.556	.389	.[a]
	Sig. (2-tailed)	.199	.781	.646	.045	.	.033	.010	.030	.011		.118	.031	.037	.006	.015	.040	.005	.151	.087	.120	.301	.
	N	9	9	9	9	0	9	9	9	9	9	9	9	9	9	9	9	9	9	9	9	9	0
Thr	Pearson Correlation	-.065	-.226	.546	.398	.[a]	.952**	.868**	.951**	.928**	.558	1	.947**	.960**	.756*	.874**	.801**	.218	.166	.977**	-.227	.780*	.[a]
	Sig. (2-tailed)	.869	.559	.128	.289	.	<.001	.002	<.001	<.001	.118		<.001	<.001	.018	.002	.009	.574	.670	<.001	.557	.013	.
	N	9	9	9	9	0	9	9	9	9	9	9	9	9	9	9	9	9	9	9	9	9	0
Val	Pearson Correlation	.167	-.259	.413	.494	.[a]	.967**	.942**	.994**	.978**	.715*	.947**	1	.988**	.827**	.901**	.863**	.451	.453	.980**	-.061	.747*	.[a]
	Sig. (2-tailed)	.667	.501	.270	.177	.	<.001	<.001	<.001	<.001	.031	<.001		<.001	.006	<.001	.003	.224	.221	<.001	.876	.021	.
	N	9	9	9	9	0	9	9	9	9	9	9	9	9	9	9	9	9	9	9	9	9	0
Ala	Pearson Correlation	.144	-.253	.456	.496	.[a]	.990**	.957**	.997**	.985**	.696*	.960**	.988**	1	.861**	.925**	.904**	.445	.402	.989**	-.090	.818**	.[a]
	Sig. (2-tailed)	.711	.511	.217	.174	.	<.001	<.001	<.001	<.001	.037	<.001	<.001		.003	<.001	<.001	.230	.284	<.001	.817	.007	.
	N	9	9	9	9	0	9	9	9	9	9	9	9	9	9	9	9	9	9	9	9	9	0
Arg	Pearson Correlation	.467	-.272	.259	.805**	.[a]	.879**	.917**	.872**	.900**	.829**	.756*	.827**	.861**	1	.925**	.804**	.728*	.386	.796*	.159	.693*	.[a]
	Sig. (2-tailed)	.205	.478	.501	.009	.	.002	<.001	.002	<.001	.006	.018	.006	.003		<.001	.009	.026	.304	.010	.683	.039	.
	N	9	9	9	9	0	9	9	9	9	9	9	9	9	9	9	9	9	9	9	9	9	0
Asp	Pearson Correlation	.153	-.152	.237	.582	.[a]	.928**	.884**	.926**	.924**	.770*	.874**	.901**	.925**	.925**	1	.842**	.498	.276	.890**	.162	.766*	.[a]

	Sig. (2-tailed)	.695	.695	.540	.100	.	<.001	.002	<.001	<.001	.015	.002	<.001	<.001	<.001		.004	.173	.472	.001	.678	.016	.
	N	9	9	9	9	0	9	9	9	9	9	9	9	9	9	9	9	9	9	9	9	9	0
Glu	Pearson Correlation	.129	-.082	.283	.374	a.	.891**	.887**	.888**	.888**	.690*	.801**	.863**	.904**	.804**	.842**	1	.527	.522	.854**	.086	.890**	a.
	Sig. (2-tailed)	.742	.833	.461	.321	.	.001	.001	.001	.001	.040	.009	.003	<.001	.009	.004		.145	.150	.003	.825	.001	.
	N	9	9	9	9	0	9	9	9	9	9	9	9	9	9	9	9	9	9	9	9	9	0
Gly	Pearson Correlation	.838**	-.078	-.152	.769*	a.	.463	.638	.476	.558	.837**	.218	.451	.445	.728*	.498	.527	1	.715*	.329	.492	.225	a.
	Sig. (2-tailed)	.005	.841	.695	.016	.	.210	.065	.196	.118	.005	.574	.224	.230	.026	.173	.145		.030	.387	.179	.560	.
	N	9	9	9	9	0	9	9	9	9	9	9	9	9	9	9	9	9	9	9	9	9	0
Pro	Pearson Correlation	.640	-.155	-.023	.308	a.	.359	.493	.430	.436	.520	.166	.453	.402	.386	.276	.522	.715*	1	.344	.242	.231	a.
	Sig. (2-tailed)	.063	.690	.952	.421	.	.343	.178	.248	.241	.151	.670	.221	.284	.304	.472	.150	.030		.365	.531	.549	.
	N	9	9	9	9	0	9	9	9	9	9	9	9	9	9	9	9	9	9	9	9	9	0
Ser	Pearson Correlation	.069	-.283	.538	.420	a.	.979**	.918**	.982**	.958**	.601	.977**	.980**	.989**	.796*	.890**	.854**	.329	.344	1	-.202	.800**	a.
	Sig. (2-tailed)	.860	.460	.135	.260	.	<.001	<.001	<.001	<.001	.087	<.001	<.001	<.001	.010	.001	.003	.387	.365		.603	.010	.
	N	9	9	9	9	0	9	9	9	9	9	9	9	9	9	9	9	9	9	9	9	9	0
Tyr	Pearson Correlation	.184	.569	-.865**	.158	a.	-.090	-.011	-.072	.002	.556	-.227	-.061	-.090	.159	.162	.086	.492	.242	-.202	1	-.181	a.
	Sig. (2-tailed)	.636	.110	.003	.684	.	.817	.977	.855	.996	.120	.557	.876	.817	.683	.678	.825	.179	.531	.603		.641	.
	N	9	9	9	9	0	9	9	9	9	9	9	9	9	9	9	9	9	9	9	9	9	0
Asn	Pearson Correlation	-.088	-.320	.421	.234	a.	.797*	.758*	.792*	.746*	.389	.780*	.747*	.818**	.693*	.766*	.890**	.225	.231	.800**	-.181	1	a.
	Sig. (2-tailed)	.821	.401	.260	.545	.	.010	.018	.011	.021	.301	.013	.021	.007	.039	.016	.001	.560	.549	.010	.641		.
	N	9	9	9	9	0	9	9	9	9	9	9	9	9	9	9	9	9	9	9	9	9	0
Gln	Pearson Correlation	a.	a.	a.	a.	a.	a.	a.	a.	a.	a.	a.	a.	a.	a.	a.	a.	a.	a.	a.	a.	a.	a.
	Sig. (2-tailed)	.	.	.	.	.	.	.	.	.	.	.	.	.	.	.	.	.	.	.	.	.	
	N	0	0	0	0	0	0	0	0	0	0	0	0	0	0	0	0	0	0	0	0	0	0

**. Correlation is significant at the 0.01 level (2-tailed).

*. Correlation is significant at the 0.05 level (2-tailed).

a. Cannot be computed because at least one of the variables is constant.

Confidence Intervals

	Pearson Correlation	Sig. (2-tailed)	95% Confidence Intervals (2-tailed)[a]	
			Lower	Upper
FRAP - ORAC	-.443	.098	-.772	.105
FRAP - DPP_IV	-.103	.715	-.582	.435
FRAP - DH	.420	.119	-.133	.761
FRAP - His	.[b]	.	.	.
FRAP - Ile	.163	.676	-.569	.742
FRAP - Leu	.357	.346	-.421	.818
FRAP - Lys	.191	.622	-.550	.754
FRAP - Met	.248	.520	-.510	.777
FRAP - Phe	.473	.199	-.306	.858
FRAP - Thr	-.065	.869	-.697	.629
FRAP - Val	.167	.667	-.566	.744
FRAP - Ala	.144	.711	-.581	.734
FRAP - Arg	.467	.205	-.312	.856
FRAP - Asp	.153	.695	-.576	.737
FRAP - Glu	.129	.742	-.591	.727
FRAP - Gly	.838	.005	.346	.961
FRAP - Pro	.640	.063	-.082	.908
FRAP - Ser	.069	.860	-.626	.699

FRAP - Tyr	.184	.636	-.555	.751
FRAP - Asn	-.088	.821	-.708	.615
FRAP - Gln	.[b]	.	.	.
ORAC - DPP_IV	-.366	.180	-.734	.193
ORAC - DH	-.169	.547	-.623	.381
ORAC - His	.[b]	.	.	.
ORAC - Ile	-.208	.592	-.761	.539
ORAC - Leu	-.288	.453	-.793	.479
ORAC - Lys	-.284	.458	-.791	.482
ORAC - Met	-.196	.614	-.756	.547
ORAC - Phe	.108	.781	-.603	.717
ORAC - Thr	-.226	.559	-.768	.526
ORAC - Val	-.259	.501	-.782	.501
ORAC - Ala	-.253	.511	-.779	.506
ORAC - Arg	-.272	.478	-.787	.491
ORAC - Asp	-.152	.695	-.737	.576
ORAC - Glu	-.082	.833	-.705	.619
ORAC - Gly	-.078	.841	-.703	.621
ORAC - Pro	-.155	.690	-.738	.574
ORAC - Ser	-.283	.460	-.791	.483
ORAC - Tyr	.569	.110	-.188	.888
ORAC - Asn	-.320	.401	-.805	.453
ORAC - Gln	.[b]	.	.	.
DPP_IV - DH	-.018	.948	-.525	.499

DPP_IV - His	.[b]	.	.	.
DPP_IV - Ile	.470	.202	-.309	.857
DPP_IV - Leu	.372	.324	-.408	.824
DPP_IV - Lys	.441	.235	-.340	.847
DPP_IV - Met	.389	.301	-.392	.829
DPP_IV - Phe	-.178	.646	-.748	.559
DPP_IV - Thr	.546	.128	-.218	.881
DPP_IV - Val	.413	.270	-.369	.838
DPP_IV - Ala	.456	.217	-.324	.852
DPP_IV - Arg	.259	.501	-.501	.781
DPP_IV - Asp	.237	.540	-.518	.772
DPP_IV - Glu	.283	.461	-.483	.791
DPP_IV - Gly	-.152	.695	-.737	.576
DPP_IV - Pro	-.023	.952	-.676	.652
DPP_IV - Ser	.538	.135	-.229	.878
DPP_IV - Tyr	-.865	.003	-.968	-.430
DPP_IV - Asn	.421	.260	-.361	.840
DPP_IV - Gln	.[b]	.	.	.
DH - His	.[b]	.	.	.
DH - Ile	.514	.157	-.258	.871
DH - Leu	.620	.075	-.113	.903
DH - Lys	.539	.134	-.227	.879
DH - Met	.583	.099	-.168	.892
DH - Phe	.678	.045	-.018	.919
DH - Thr	.398	.289	-.383	.833

DH - Val	.494	.177	-.282	.864
DH - Ala	.496	.174	-.280	.865
DH - Arg	.805	.009	.256	.953
DH - Asp	.582	.100	-.169	.892
DH - Glu	.374	.321	-.406	.824
DH - Gly	.769	.016	.167	.944
DH - Pro	.308	.421	-.463	.800
DH - Ser	.420	.260	-.361	.840
DH - Tyr	.158	.684	-.572	.740
DH - Asn	.234	.545	-.520	.771
DH - Gln	.[b]	.	.	.
His - Ile	.[b]	.	.	.
His - Leu	.[b]	.	.	.
His - Lys	.[b]	.	.	.
His - Met	.[b]	.	.	.
His - Phe	.[b]	.	.	.
His - Thr	.[b]	.	.	.
His - Val	.[b]	.	.	.
His - Ala	.[b]	.	.	.
His - Arg	.[b]	.	.	.
His - Asp	.[b]	.	.	.
His - Glu	.[b]	.	.	.
His - Gly	.[b]	.	.	.
His - Pro	.[b]	.	.	.
His - Ser	.[b]	.	.	.

His - Tyr	.[b]	.	.	.
His - Asn	.[b]	.	.	.
His - Gln	.[b]	.	.	.
Ile - Leu	.962	<.001	.805	.991
Ile - Lys	.982	<.001	.902	.996
Ile - Met	.987	<.001	.930	.997
Ile - Phe	.708	.033	.040	.927
Ile - Thr	.952	<.001	.760	.989
Ile - Val	.967	<.001	.828	.992
Ile - Ala	.990	<.001	.947	.998
Ile - Arg	.879	.002	.476	.971
Ile - Asp	.928	<.001	.657	.983
Ile - Glu	.891	.001	.516	.974
Ile - Gly	.463	.210	-.317	.854
Ile - Pro	.359	.343	-.420	.819
Ile - Ser	.979	<.001	.886	.995
Ile - Tyr	-.090	.817	-.709	.614
Ile - Asn	.797	.010	.237	.951
Ile - Gln	.[b]	.	.	.
Leu - Lys	.958	<.001	.787	.990
Leu - Met	.980	<.001	.894	.995
Leu - Phe	.798	.010	.239	.951
Leu - Thr	.868	.002	.438	.969
Leu - Val	.942	<.001	.715	.987
Leu - Ala	.957	<.001	.781	.990

Leu - Arg	.917	<.001	.613	.981
Leu - Asp	.884	.002	.493	.973
Leu - Glu	.887	.001	.501	.973
Leu - Gly	.638	.065	-.085	.908
Leu - Pro	.493	.178	-.283	.864
Leu - Ser	.918	<.001	.616	.981
Leu - Tyr	-.011	.977	-.670	.658
Leu - Asn	.758	.018	.143	.941
Leu - Gln	.[b]	.	.	.
Lys - Met	.986	<.001	.924	.997
Lys - Phe	.715	.030	.053	.929
Lys - Thr	.951	<.001	.752	.989
Lys - Val	.994	<.001	.965	.999
Lys - Ala	.997	<.001	.982	.999
Lys - Arg	.872	.002	.450	.970
Lys - Asp	.926	<.001	.646	.983
Lys - Glu	.888	.001	.505	.974
Lys - Gly	.476	.196	-.303	.859
Lys - Pro	.430	.248	-.352	.843
Lys - Ser	.982	<.001	.906	.996
Lys - Tyr	-.072	.855	-.700	.625
Lys - Asn	.792	.011	.223	.950
Lys - Gln	.[b]	.	.	.
Met - Phe	.793	.011	.225	.950
Met - Thr	.928	<.001	.655	.983

Met - Val	.978	<.001	.882	.995
Met - Ala	.985	<.001	.920	.997
Met - Arg	.900	<.001	.550	.977
Met - Asp	.924	<.001	.642	.982
Met - Glu	.888	.001	.505	.974
Met - Gly	.558	.118	-.202	.884
Met - Pro	.436	.241	-.345	.846
Met - Ser	.958	<.001	.788	.990
Met - Tyr	.002	.996	-.663	.665
Met - Asn	.746	.021	.116	.937
Met - Gln	.[b]	.	.	.
Phe - Thr	.558	.118	-.202	.884
Phe - Val	.715	.031	.052	.929
Phe - Ala	.696	.037	.016	.924
Phe - Arg	.829	.006	.322	.959
Phe - Asp	.770	.015	.170	.944
Phe - Glu	.690	.040	.004	.922
Phe - Gly	.837	.005	.344	.961
Phe - Pro	.520	.151	-.251	.873
Phe - Ser	.601	.087	-.142	.897
Phe - Tyr	.556	.120	-.205	.884
Phe - Asn	.389	.301	-.392	.829
Phe - Gln	.[b]	.	.	.
Thr - Val	.947	<.001	.735	.988
Thr - Ala	.960	<.001	.795	.991

Thr - Arg	.756	.018	.139	.940
Thr - Asp	.874	.002	.459	.970
Thr - Glu	.801	.009	.246	.952
Thr - Gly	.218	.574	-.532	.765
Thr - Pro	.166	.670	-.567	.743
Thr - Ser	.977	<.001	.876	.995
Thr - Tyr	-.227	.557	-.769	.525
Thr - Asn	.780	.013	.193	.946
Thr - Gln	.[b]	.	.	.
Val - Ala	.988	<.001	.935	.997
Val - Arg	.827	.006	.316	.959
Val - Asp	.901	<.001	.552	.977
Val - Glu	.863	.003	.423	.967
Val - Gly	.451	.224	-.330	.850
Val - Pro	.453	.221	-.328	.851
Val - Ser	.980	<.001	.892	.995
Val - Tyr	-.061	.876	-.695	.631
Val - Asn	.747	.021	.118	.938
Val - Gln	.[b]	.	.	.
Ala - Arg	.861	.003	.416	.967
Ala - Asp	.925	<.001	.643	.982
Ala - Glu	.904	<.001	.562	.977
Ala - Gly	.445	.230	-.336	.849
Ala - Pro	.402	.284	-.379	.834
Ala - Ser	.989	<.001	.937	.997

Ala - Tyr	-.090	.817	-.709	.614
Ala - Asn	.818	.007	.291	.956
Ala - Gln	.[b]	.	.	.
Arg - Asp	.925	<.001	.643	.982
Arg - Glu	.804	.009	.254	.953
Arg - Gly	.728	.026	.080	.933
Arg - Pro	.386	.304	-.394	.829
Arg - Ser	.796	.010	.234	.951
Arg - Tyr	.159	.683	-.571	.740
Arg - Asn	.693	.039	.010	.923
Arg - Gln	.[b]	.	.	.
Asp - Glu	.842	.004	.359	.962
Asp - Gly	.498	.173	-.277	.866
Asp - Pro	.276	.472	-.488	.788
Asp - Ser	.890	.001	.514	.974
Asp - Tyr	.162	.678	-.570	.741
Asp - Asn	.766	.016	.160	.943
Asp - Gln	.[b]	.	.	.
Glu - Gly	.527	.145	-.242	.875
Glu - Pro	.522	.150	-.249	.873
Glu - Ser	.854	.003	.396	.965
Glu - Tyr	.086	.825	-.616	.707
Glu - Asn	.890	.001	.514	.974
Glu - Gln	.[b]	.	.	.
Gly - Pro	.715	.030	.053	.929

Gly - Ser	.329	.387	-.445	.808
Gly - Tyr	.492	.179	-.285	.864
Gly - Asn	.225	.560	-.526	.768
Gly - Gln	.[b]	.	.	.
Pro - Ser	.344	.365	-.433	.813
Pro - Tyr	.242	.531	-.514	.775
Pro - Asn	.231	.549	-.522	.770
Pro - Gln	.[b]	.	.	.
Ser - Tyr	-.202	.603	-.758	.543
Ser - Asn	.800	.010	.243	.952
Ser - Gln	.[b]	.	.	.
Tyr - Asn	-.181	.641	-.749	.557
Tyr - Gln	.[b]	.	.	.
Asn - Gln	.[b]	.	.	.

a. Estimation is based on Fisher's r-to-z transformation with bias adjustment.

b. Cannot be computed because at least one of the variables is constant.
