# Supplementary material for: In Vitro Gastrointestinal Digestion of Calanus finmarchicus Products: Amino Acid Composition, Degree of Hydrolysis, Antioxidant Capacity, and Antidiabetic Activity
Source: Mar Drugs. 2026 Jul 7;24(7):240. doi: 10.3390/md24070240 (PMC13412531; doi:10.3390/md24070240)
Supplement: Supplementary file 1 [file marinedrugs-24-00240-s001.zip › Table_S5_FDCF_Pearson-Correlation-Coefficients.pdf]

Table S5. Pearson correlation matrices (r) for freeze-dried *C. finmarchicus* (FDCF) across FRAP, ORAC, DPP-IV, DH, and individual FAA.

Descriptive Statistics

	Mean	Std. Deviation	N
FRAP	8.6700	2.19592	15
ORAC	304.1193	71.77429	15
DPP_IV	5.2810	5.33815	15
DH	3.6153	5.10603	15
His	.14023	.018255	9
Ile	.42066	.043162	9
Leu	.84289	.153975	9
Lys	.88591	.133649	9
Met	.29756	.039076	9
Phe	.47621	.093036	9
Thr	.36323	.055271	9
Val	.53552	.066330	9
Ala	.65043	.086518	9
Arg	1.40920	.118361	9
Asp	.25919	.032058	9
Glu	.48208	.059451	9
Gly	.78201	.149783	9

Pro	.34913	.057307	9
Ser	.30493	.032679	9
Tyr	.43787	.194614	9
Asn	.51543	.053616	9
Gln	.20533	.039888	9

Correlations

		FRAP	ORAC	DPP_IV	DH	His	Ile	Leu	Lys	Met	Phe	Thr	Val	Ala	Arg	Asp	Glu	Gly	Pro	Ser	Tyr	Asn	Gln
FRAP	Pearson Correlation	1	.820**	-.061	.250	-.202	.387	.772*	.290	.345	.807**	.276	.302	.273	.018	.129	.534	.313	-.082	.057	.917**	.043	.238
	Sig. (2-tailed)		<.001	.830	.369	.603	.304	.015	.448	.363	.009	.472	.430	.478	.964	.741	.138	.412	.835	.884	<.001	.912	.538
	N	15	15	15	15	9	9	9	9	9	9	9	9	9	9	9	9	9	9	9	9	9	9
ORAC	Pearson Correlation	.820**	1	-.030	.624*	-.178	.335	.654	.232	.094	.759*	.375	.456	.232	.068	.073	.478	.076	-.038	.047	.929**	.061	-.079
	Sig. (2-tailed)	<.001		.916	.013	.647	.378	.056	.547	.810	.018	.320	.218	.548	.862	.851	.193	.845	.923	.904	<.001	.877	.840
	N	15	15	15	15	9	9	9	9	9	9	9	9	9	9	9	9	9	9	9	9	9	9
DPP_IV	Pearson Correlation	-.061	-.030	1	-.121	-.018	.035	.075	.018	.250	.055	-.101	-.367	-.001	-.196	.002	.058	.105	-.048	.006	.012	-.057	.127
	Sig. (2-tailed)	.830	.916		.667	.964	.928	.849	.963	.517	.888	.796	.331	.998	.614	.996	.882	.788	.902	.988	.976	.885	.746
	N	15	15	15	15	9	9	9	9	9	9	9	9	9	9	9	9	9	9	9	9	9	9
DH	Pearson Correlation	.250	.624*	-.121	1	-.387	-.231	-.047	-.247	-.428	.070	-.096	.019	-.262	-.252	-.395	-.131	-.444	-.308	-.381	.322	-.264	-.517
	Sig. (2-tailed)	.369	.013	.667		.303	.549	.905	.522	.251	.858	.806	.961	.496	.513	.292	.736	.231	.421	.312	.398	.493	.154
	N	15	15	15	15	9	9	9	9	9	9	9	9	9	9	9	9	9	9	9	9	9	9
His	Pearson Correlation	-.202	-.178	-.018	-.387	1	.787*	.419	.807**	.476	.381	.794*	.736*	.844**	.644	.917**	.687*	.626	.875**	.927**	.022	.908**	.443
	Sig. (2-tailed)	.603	.647	.964	.303		.012	.262	.009	.195	.312	.011	.024	.004	.061	<.001	.041	.071	.002	<.001	.954	<.001	.232
	N	9	9	9	9	9	9	9	9	9	9	9	9	9	9	9	9	9	9	9	9	9	9
Ile	Pearson Correlation	.387	.335	.035	-.231	.787*	1	.856**	.940**	.770*	.834**	.892**	.873**	.974**	.748*	.956**	.971**	.811**	.866**	.924**	.564	.923**	.608
	Sig. (2-tailed)	.304	.378	.928	.549	.012		.003	<.001	.015	.005	.001	.002	<.001	.020	<.001	<.001	.008	.003	<.001	.113	<.001	.082

	N	9	9	9	9	9	9	9	9	9	9	9	9	9	9	9	9	9	9	9	9	9	9
Leu	Pearson Correlation	.772*	.654	.075	-.047	.419	.856**	1	.830**	.625	.967**	.762*	.743*	.817**	.412	.699*	.893**	.766*	.533	.613	.853**	.626	.604
	Sig. (2-tailed)	.015	.056	.849	.905	.262	.003		.006	.072	<.001	.017	.022	.007	.271	.036	.001	.016	.140	.079	.003	.071	.085
	N	9	9	9	9	9	9	9	9	9	9	9	9	9	9	9	9	9	9	9	9	9	9
Lys	Pearson Correlation	.290	.232	.018	-.247	.807**	.940**	.830**	1	.631	.739*	.885**	.835**	.988**	.563	.932**	.868**	.897**	.851**	.854**	.466	.896**	.730*
	Sig. (2-tailed)	.448	.547	.963	.522	.009	<.001	.006		.068	.023	.002	.005	<.001	.114	<.001	.002	.001	.004	.003	.206	.001	.025
	N	9	9	9	9	9	9	9	9	9	9	9	9	9	9	9	9	9	9	9	9	9	9
Met	Pearson Correlation	.345	.094	.250	-.428	.476	.770*	.625	.631	1	.581	.422	.471	.689*	.784*	.713*	.759*	.738*	.688*	.685*	.326	.724*	.684*
	Sig. (2-tailed)	.363	.810	.517	.251	.195	.015	.072	.068		.101	.258	.201	.040	.012	.031	.018	.023	.041	.042	.393	.027	.042
	N	9	9	9	9	9	9	9	9	9	9	9	9	9	9	9	9	9	9	9	9	9	9
Phe	Pearson Correlation	.807**	.759*	.055	.070	.381	.834**	.967**	.739*	.581	1	.769*	.761*	.753*	.450	.653	.907**	.608	.481	.609	.920**	.584	.404
	Sig. (2-tailed)	.009	.018	.888	.858	.312	.005	<.001	.023	.101		.015	.017	.019	.224	.056	<.001	.082	.190	.082	<.001	.099	.281
	N	9	9	9	9	9	9	9	9	9	9	9	9	9	9	9	9	9	9	9	9	9	9
Thr	Pearson Correlation	.276	.375	-.101	-.096	.794*	.892**	.762*	.885**	.422	.769*	1	.911**	.905**	.573	.879**	.844**	.625	.795*	.874**	.561	.810**	.368
	Sig. (2-tailed)	.472	.320	.796	.806	.011	.001	.017	.002	.258	.015		<.001	<.001	.107	.002	.004	.072	.010	.002	.116	.008	.330
	N	9	9	9	9	9	9	9	9	9	9	9	9	9	9	9	9	9	9	9	9	9	9
Val	Pearson Correlation	.302	.456	-.367	.019	.736*	.873**	.743*	.835**	.471	.761*	.911**	1	.876**	.679*	.834**	.864**	.632	.758*	.829**	.551	.845**	.400
	Sig. (2-tailed)	.430	.218	.331	.961	.024	.002	.022	.005	.201	.017	<.001		.002	.044	.005	.003	.068	.018	.006	.124	.004	.286
	N	9	9	9	9	9	9	9	9	9	9	9	9	9	9	9	9	9	9	9	9	9	9
Ala	Pearson Correlation	.273	.232	-.001	-.262	.844**	.974**	.817**	.988**	.689*	.753*	.905**	.876**	1	.671*	.967**	.912**	.871**	.893**	.916**	.462	.942**	.687*
	Sig. (2-tailed)	.478	.548	.998	.496	.004	<.001	.007	<.001	.040	.019	<.001	.002		.048	<.001	<.001	.002	.001	<.001	.211	<.001	.041
	N	9	9	9	9	9	9	9	9	9	9	9	9	9	9	9	9	9	9	9	9	9	9
Arg	Pearson Correlation	.018	.068	-.196	-.252	.644	.748*	.412	.563	.784*	.450	.573	.679*	.671*	1	.763*	.702*	.482	.827**	.818**	.188	.811**	.338
	Sig. (2-tailed)	.964	.862	.614	.513	.061	.020	.271	.114	.012	.224	.107	.044	.048		.017	.035	.189	.006	.007	.628	.008	.374
	N	9	9	9	9	9	9	9	9	9	9	9	9	9	9	9	9	9	9	9	9	9	9
Asp	Pearson Correlation	.129	.073	.002	-.395	.917**	.956**	.699*	.932**	.713*	.653	.879**	.834**	.967**	.763*	1	.877**	.799**	.934**	.978**	.322	.960**	.612

	Sig. (2-tailed)	.741	.851	.996	.292	<.001	<.001	.036	<.001	.031	.056	.002	.005	<.001	.017		.002	.010	<.001	<.001	.397	<.001	.080
	N	9	9	9	9	9	9	9	9	9	9	9	9	9	9	9	9	9	9	9	9	9	9
Glu	Pearson Correlation	.534	.478	.058	-.131	.687*	.971**	.893**	.868**	.759*	.907**	.844**	.864**	.912**	.702*	.877**	1	.742*	.741*	.855**	.677*	.853**	.530
	Sig. (2-tailed)	.138	.193	.882	.736	.041	<.001	.001	.002	.018	<.001	.004	.003	<.001	.035	.002		.022	.022	.003	.045	.003	.142
	N	9	9	9	9	9	9	9	9	9	9	9	9	9	9	9	9	9	9	9	9	9	9
Gly	Pearson Correlation	.313	.076	.105	-.444	.626	.811**	.766*	.897**	.738*	.608	.625	.632	.871**	.482	.799**	.742*	1	.720*	.687*	.331	.782*	.952**
	Sig. (2-tailed)	.412	.845	.788	.231	.071	.008	.016	.001	.023	.082	.072	.068	.002	.189	.010	.022		.029	.041	.384	.013	<.001
	N	9	9	9	9	9	9	9	9	9	9	9	9	9	9	9	9	9	9	9	9	9	9
Pro	Pearson Correlation	-.082	-.038	-.048	-.308	.875**	.866**	.533	.851**	.688*	.481	.795*	.758*	.893**	.827**	.934**	.741*	.720*	1	.930**	.163	.947**	.557
	Sig. (2-tailed)	.835	.923	.902	.421	.002	.003	.140	.004	.041	.190	.010	.018	.001	.006	<.001	.022	.029		<.001	.675	<.001	.119
	N	9	9	9	9	9	9	9	9	9	9	9	9	9	9	9	9	9	9	9	9	9	9
Ser	Pearson Correlation	.057	.047	.006	-.381	.927**	.924**	.613	.854**	.685*	.609	.874**	.829**	.916**	.818**	.978**	.855**	.687*	.930**	1	.279	.948**	.476
	Sig. (2-tailed)	.884	.904	.988	.312	<.001	<.001	.079	.003	.042	.082	.002	.006	<.001	.007	<.001	.003	.041	<.001		.468	<.001	.195
	N	9	9	9	9	9	9	9	9	9	9	9	9	9	9	9	9	9	9	9	9	9	9
Tyr	Pearson Correlation	.917**	.929**	.012	.322	.022	.564	.853**	.466	.326	.920**	.561	.551	.462	.188	.322	.677*	.331	.163	.279	1	.244	.157
	Sig. (2-tailed)	<.001	<.001	.976	.398	.954	.113	.003	.206	.393	<.001	.116	.124	.211	.628	.397	.045	.384	.675	.468		.526	.687
	N	9	9	9	9	9	9	9	9	9	9	9	9	9	9	9	9	9	9	9	9	9	9
Asn	Pearson Correlation	.043	.061	-.057	-.264	.908**	.923**	.626	.896**	.724*	.584	.810**	.845**	.942**	.811**	.960**	.853**	.782*	.947**	.948**	.244	1	.608
	Sig. (2-tailed)	.912	.877	.885	.493	<.001	<.001	.071	.001	.027	.099	.008	.004	<.001	.008	<.001	.003	.013	<.001	<.001	.526		.082
	N	9	9	9	9	9	9	9	9	9	9	9	9	9	9	9	9	9	9	9	9	9	9
Gln	Pearson Correlation	.238	-.079	.127	-.517	.443	.608	.604	.730*	.684*	.404	.368	.400	.687*	.338	.612	.530	.952**	.557	.476	.157	.608	1
	Sig. (2-tailed)	.538	.840	.746	.154	.232	.082	.085	.025	.042	.281	.330	.286	.041	.374	.080	.142	<.001	.119	.195	.687	.082	
	N	9	9	9	9	9	9	9	9	9	9	9	9	9	9	9	9	9	9	9	9	9	9

**. Correlation is significant at the 0.01 level (2-tailed).

*. Correlation is significant at the 0.05 level (2-tailed).

Confidence Intervals

	Pearson Correlation	Sig. (2-tailed)	95% Confidence Intervals (2-tailed)[a]	
			Lower	Upper
FRAP - ORAC	.820	<.001	.508	.934
FRAP - DPP_IV	-.061	.830	-.554	.468
FRAP - DH	.250	.369	-.309	.671
FRAP - His	-.202	.603	-.758	.543
FRAP - Ile	.387	.304	-.394	.829
FRAP - Leu	.772	.015	.176	.944
FRAP - Lys	.290	.448	-.477	.794
FRAP - Met	.345	.363	-.432	.814
FRAP - Phe	.807	.009	.260	.953
FRAP - Thr	.276	.472	-.489	.788
FRAP - Val	.302	.430	-.468	.798
FRAP - Ala	.273	.478	-.491	.787
FRAP - Arg	.018	.964	-.655	.673
FRAP - Asp	.129	.741	-.590	.727
FRAP - Glu	.534	.138	-.233	.877
FRAP - Gly	.313	.412	-.459	.802
FRAP - Pro	-.082	.835	-.705	.619
FRAP - Ser	.057	.884	-.633	.693
FRAP - Tyr	.917	<.001	.611	.980
FRAP - Asn	.043	.912	-.641	.686

FRAP - Gln	.238	.538	-.517	.773
ORAC - DPP_IV	-.030	.916	-.533	.491
ORAC - DH	.624	.013	.142	.855
ORAC - His	-.178	.647	-.748	.559
ORAC - Ile	.335	.378	-.440	.810
ORAC - Leu	.654	.056	-.059	.912
ORAC - Lys	.232	.547	-.521	.771
ORAC - Met	.094	.810	-.612	.711
ORAC - Phe	.759	.018	.144	.941
ORAC - Thr	.375	.320	-.405	.825
ORAC - Val	.456	.218	-.324	.852
ORAC - Ala	.232	.548	-.522	.771
ORAC - Arg	.068	.862	-.627	.698
ORAC - Asp	.073	.851	-.624	.701
ORAC - Glu	.478	.193	-.300	.859
ORAC - Gly	.076	.845	-.622	.702
ORAC - Pro	-.038	.923	-.684	.644
ORAC - Ser	.047	.904	-.638	.688
ORAC - Tyr	.929	<.001	.661	.983
ORAC - Asn	.061	.877	-.631	.695
ORAC - Gln	-.079	.840	-.704	.621
DPP_IV - DH	-.121	.667	-.594	.420
DPP_IV - His	-.018	.964	-.673	.655
DPP_IV - Ile	.035	.928	-.645	.682
DPP_IV - Leu	.075	.849	-.623	.701

DPP_IV - Lys	.018	.963	-.654	.674
DPP_IV - Met	.250	.517	-.508	.778
DPP_IV - Phe	.055	.888	-.634	.692
DPP_IV - Thr	-.101	.796	-.714	.608
DPP_IV - Val	-.367	.331	-.822	.412
DPP_IV - Ala	-.001	.998	-.665	.663
DPP_IV - Arg	-.196	.614	-.756	.547
DPP_IV - Asp	.002	.996	-.663	.665
DPP_IV - Glu	.058	.882	-.633	.693
DPP_IV - Gly	.105	.788	-.605	.716
DPP_IV - Pro	-.048	.902	-.689	.638
DPP_IV - Ser	.006	.988	-.661	.667
DPP_IV - Tyr	.012	.976	-.658	.670
DPP_IV - Asn	-.057	.885	-.693	.633
DPP_IV - Gln	.127	.746	-.592	.726
DH - His	-.387	.303	-.829	.394
DH - Ile	-.231	.549	-.770	.522
DH - Leu	-.047	.905	-.688	.639
DH - Lys	-.247	.522	-.777	.511
DH - Met	-.428	.251	-.843	.354
DH - Phe	.070	.858	-.626	.699
DH - Thr	-.096	.806	-.712	.611
DH - Val	.019	.961	-.654	.674
DH - Ala	-.262	.496	-.783	.499
DH - Arg	-.252	.513	-.779	.507
DH - Asp	-.395	.292	-.832	.386

DH - Glu	-.131	.736	-.728	.589
DH - Gly	-.444	.231	-.848	.337
DH - Pro	-.308	.421	-.800	.463
DH - Ser	-.381	.312	-.827	.400
DH - Tyr	.322	.398	-.451	.806
DH - Asn	-.264	.493	-.783	.498
DH - Gln	-.517	.154	-.872	.255
His - Ile	.787	.012	.211	.948
His - Leu	.419	.262	-.363	.840
His - Lys	.807	.009	.261	.953
His - Met	.476	.195	-.302	.859
His - Phe	.381	.312	-.399	.827
His - Thr	.794	.011	.227	.950
His - Val	.736	.024	.096	.935
His - Ala	.844	.004	.364	.963
His - Arg	.644	.061	-.075	.910
His - Asp	.917	<.001	.614	.981
His - Glu	.687	.041	-.001	.922
His - Gly	.626	.071	-.103	.905
His - Pro	.875	.002	.460	.970
His - Ser	.927	<.001	.652	.983
His - Tyr	.022	.954	-.652	.676
His - Asn	.908	<.001	.577	.978
His - Gln	.443	.232	-.338	.848
Ile - Leu	.856	.003	.401	.966
Ile - Lys	.940	<.001	.705	.986

Ile - Met	.770	.015	.171	.944
Ile - Phe	.834	.005	.336	.960
Ile - Thr	.892	.001	.519	.975
Ile - Val	.873	.002	.455	.970
Ile - Ala	.974	<.001	.862	.994
Ile - Arg	.748	.020	.121	.938
Ile - Asp	.956	<.001	.777	.990
Ile - Glu	.971	<.001	.848	.993
Ile - Gly	.811	.008	.271	.954
Ile - Pro	.866	.003	.434	.968
Ile - Ser	.924	<.001	.641	.982
Ile - Tyr	.564	.113	-.194	.886
Ile - Asn	.923	<.001	.635	.982
Ile - Gln	.608	.082	-.131	.899
Leu - Lys	.830	.006	.324	.959
Leu - Met	.625	.072	-.105	.904
Leu - Phe	.967	<.001	.829	.992
Leu - Thr	.762	.017	.152	.942
Leu - Val	.743	.022	.111	.937
Leu - Ala	.817	.007	.288	.956
Leu - Arg	.412	.271	-.370	.837
Leu - Asp	.699	.036	.021	.925
Leu - Glu	.893	.001	.523	.975
Leu - Gly	.766	.016	.160	.943
Leu - Pro	.533	.140	-.235	.877
Leu - Ser	.613	.079	-.124	.901

Leu - Tyr	.853	.003	.391	.965
Leu - Asn	.626	.071	-.104	.904
Leu - Gln	.604	.085	-.137	.898
Lys - Met	.631	.068	-.095	.906
Lys - Phe	.739	.023	.102	.936
Lys - Thr	.885	.002	.494	.973
Lys - Val	.835	.005	.337	.960
Lys - Ala	.988	<.001	.934	.997
Lys - Arg	.563	.114	-.195	.886
Lys - Asp	.932	<.001	.671	.984
Lys - Glu	.868	.002	.440	.969
Lys - Gly	.897	.001	.536	.976
Lys - Pro	.851	.004	.386	.965
Lys - Ser	.854	.003	.395	.965
Lys - Tyr	.466	.206	-.313	.855
Lys - Asn	.896	.001	.535	.976
Lys - Gln	.730	.025	.084	.933
Met - Phe	.581	.101	-.171	.891
Met - Thr	.422	.258	-.359	.841
Met - Val	.471	.201	-.308	.857
Met - Ala	.689	.040	.003	.922
Met - Arg	.784	.012	.204	.948
Met - Asp	.713	.031	.049	.929
Met - Glu	.759	.018	.145	.941
Met - Gly	.738	.023	.100	.935
Met - Pro	.688	.041	.000	.922

Met - Ser	.685	.042	-.004	.921
Met - Tyr	.326	.393	-.448	.807
Met - Asn	.724	.027	.071	.932
Met - Gln	.684	.042	-.006	.921
Phe - Thr	.769	.015	.169	.944
Phe - Val	.761	.017	.149	.941
Phe - Ala	.753	.019	.132	.939
Phe - Arg	.450	.224	-.330	.850
Phe - Asp	.653	.056	-.060	.912
Phe - Glu	.907	<.001	.574	.978
Phe - Gly	.608	.082	-.131	.899
Phe - Pro	.481	.190	-.297	.860
Phe - Ser	.609	.082	-.130	.899
Phe - Tyr	.920	<.001	.622	.981
Phe - Asn	.584	.099	-.167	.892
Phe - Gln	.404	.281	-.377	.835
Thr - Val	.911	<.001	.588	.979
Thr - Ala	.905	<.001	.567	.978
Thr - Arg	.573	.107	-.183	.889
Thr - Asp	.879	.002	.474	.971
Thr - Glu	.844	.004	.363	.963
Thr - Gly	.625	.072	-.105	.904
Thr - Pro	.795	.010	.232	.950
Thr - Ser	.874	.002	.459	.970
Thr - Tyr	.561	.116	-.198	.885
Thr - Asn	.810	.008	.269	.954

Thr - Gln	.368	.330	-.412	.822
Val - Ala	.876	.002	.466	.971
Val - Arg	.679	.044	-.015	.919
Val - Asp	.834	.005	.336	.960
Val - Glu	.864	.003	.424	.968
Val - Gly	.632	.068	-.094	.906
Val - Pro	.758	.018	.142	.941
Val - Ser	.829	.006	.320	.959
Val - Tyr	.551	.124	-.212	.882
Val - Asn	.845	.004	.368	.963
Val - Gln	.400	.286	-.381	.833
Ala - Arg	.671	.048	-.029	.917
Ala - Asp	.967	<.001	.829	.992
Ala - Glu	.912	<.001	.591	.979
Ala - Gly	.871	.002	.449	.969
Ala - Pro	.893	.001	.524	.975
Ala - Ser	.916	<.001	.609	.980
Ala - Tyr	.462	.211	-.318	.854
Ala - Asn	.942	<.001	.716	.987
Ala - Gln	.687	.041	-.002	.921
Arg - Asp	.763	.017	.155	.942
Arg - Glu	.702	.035	.027	.926
Arg - Gly	.482	.189	-.296	.860
Arg - Pro	.827	.006	.317	.959
Arg - Ser	.818	.007	.291	.956
Arg - Tyr	.188	.628	-.552	.753

Arg - Asn	.811	.008	.272	.954
Arg - Gln	.338	.374	-.438	.811
Asp - Glu	.877	.002	.468	.971
Asp - Gly	.799	.010	.240	.951
Asp - Pro	.934	<.001	.680	.985
Asp - Ser	.978	<.001	.884	.995
Asp - Tyr	.322	.397	-.451	.806
Asp - Asn	.960	<.001	.797	.991
Asp - Gln	.612	.080	-.125	.900
Glu - Gly	.742	.022	.108	.936
Glu - Pro	.741	.022	.106	.936
Glu - Ser	.855	.003	.396	.965
Glu - Tyr	.677	.045	-.020	.919
Glu - Asn	.853	.003	.391	.965
Glu - Gln	.530	.142	-.238	.876
Gly - Pro	.720	.029	.062	.931
Gly - Ser	.687	.041	-.001	.922
Gly - Tyr	.331	.384	-.444	.809
Gly - Asn	.782	.013	.200	.947
Gly - Gln	.952	<.001	.761	.989
Pro - Ser	.930	<.001	.666	.984
Pro - Tyr	.163	.675	-.569	.742
Pro - Asn	.947	<.001	.738	.988
Pro - Gln	.557	.119	-.204	.884
Ser - Tyr	.279	.468	-.486	.789
Ser - Asn	.948	<.001	.739	.988

Ser - Gln	.476	.195	-.302	.859
Tyr - Asn	.244	.526	-.512	.776
Tyr - Gln	.157	.687	-.573	.739
Asn - Gln	.608	.082	-.131	.899

a. Estimation is based on Fisher's r-to-z transformation with bias adjustment.
