# Supplementary material for: In Vitro Gastrointestinal Digestion of Calanus finmarchicus Products: Amino Acid Composition, Degree of Hydrolysis, Antioxidant Capacity, and Antidiabetic Activity
Source: Mar Drugs. 2026 Jul 7;24(7):240. doi: 10.3390/md24070240 (PMC13412531; doi:10.3390/md24070240)
Supplement: Supplementary file 1 [file marinedrugs-24-00240-s001.zip › Table_S4_FFCF_Pearson-Correlation-Coefficients.pdf]

Table S4. Pearson correlation matrices (r) for fresh-frozen *C. finmarchicus* (FFCF) across FRAP, ORAC, DPP-IV, DH, and individual FAA.

Descriptive Statistics

	Mean	Std. Deviation	N
FRAP	17.5080	6.26791	15
ORAC	470.4520	191.92214	15
DPP_IV	1.53267	.671411	15
DH	4.89267	5.893863	15
His	.02709	.002580	9
Ile	.08424	.013051	9
Leu	.18659	.045723	9
Lys	.17731	.022064	9
Met	.06223	.010786	9
Phe	.14292	.057477	9
Thr	.06611	.009209	9
Val	.11137	.017775	9
Ala	.12537	.015361	9
Arg	.18464	.029600	9
Asp	.05077	.005534	9
Glu	.08469	.026700	9
Gly	.16756	.064033	9

Pro	.06534	.006409	9
Ser	.06203	.007150	9
Tyr	.14731	.044546	9
Asn	.10750	.012125	9
Gln	.03896	.009414	9

Correlations

		FRAP	ORAC	DPP_IV	DH	His	Ile	Leu	Lys	Met	Phe	Thr	Val	Ala	Arg	Asp	Glu	Gly	Pro	Ser	Tyr	Asn	Gln
FRAP	Pearson Correlation	1	.838**	-.327	.619*	.102	.202	.647	.209	.426	.857**	-.020	.328	.110	.117	.022	-.071	.748*	-.428	-.073	.903**	-.262	.415
	Sig. (2-tailed)		<.001	.235	.014	.793	.602	.060	.589	.253	.003	.960	.390	.777	.765	.955	.855	.020	.250	.852	<.001	.497	.267
	N	15	15	15	15	9	9	9	9	9	9	9	9	9	9	9	9	9	9	9	9	9	9
ORAC	Pearson Correlation	.838**	1	-.549*	.686**	.086	.352	.680*	.266	.463	.792*	.188	.434	.129	.141	.158	.179	.572	-.458	-.008	.873**	-.272	.130
	Sig. (2-tailed)	<.001		.034	.005	.826	.353	.044	.489	.209	.011	.628	.243	.741	.718	.684	.646	.108	.215	.983	.002	.479	.740
	N	15	15	15	15	9	9	9	9	9	9	9	9	9	9	9	9	9	9	9	9	9	9
DPP_IV	Pearson Correlation	-.327	-.549*	1	-.357	-.006	-.193	-.298	-.059	-.186	-.285	-.122	-.175	.062	.074	-.042	-.065	-.045	.372	.093	-.396	.187	.242
	Sig. (2-tailed)	.235	.034		.191	.988	.618	.436	.880	.632	.457	.755	.652	.874	.851	.914	.869	.909	.324	.811	.291	.629	.531
	N	15	15	15	15	9	9	9	9	9	9	9	9	9	9	9	9	9	9	9	9	9	9
DH	Pearson Correlation	.619*	.686**	-.357	1	.612	.766*	.953**	.764*	.881**	.915**	.608	.840**	.650	.698*	.608	.627	.848**	-.041	.539	.865**	.282	.646
	Sig. (2-tailed)	.014	.005	.191		.080	.016	<.001	.017	.002	<.001	.082	.005	.058	.037	.083	.071	.004	.917	.134	.003	.462	.060
	N	15	15	15	15	9	9	9	9	9	9	9	9	9	9	9	9	9	9	9	9	9	9
His	Pearson Correlation	.102	.086	-.006	.612	1	.933**	.741*	.960**	.909**	.479	.889**	.917**	.983**	.925**	.941**	.847**	.587	.715*	.958**	.327	.881**	.790*
	Sig. (2-tailed)	.793	.826	.988	.080		<.001	.022	<.001	<.001	.192	.001	<.001	<.001	<.001	<.001	.004	.097	.030	<.001	.390	.002	.011
	N	9	9	9	9	9	9	9	9	9	9	9	9	9	9	9	9	9	9	9	9	9	9
Ile	Pearson Correlation	.202	.352	-.193	.766*	.933**	1	.851**	.979**	.955**	.598	.962**	.986**	.956**	.905**	.972**	.906**	.613	.549	.901**	.487	.725*	.660
	Sig. (2-tailed)	.602	.353	.618	.016	<.001		.004	<.001	<.001	.089	<.001	<.001	<.001	<.001	<.001	<.001	.079	.126	<.001	.183	.027	.053

	N	9	9	9	9	9	9	9	9	9	9	9	9	9	9	9	9	9	9	9	9	9	9
Leu	Pearson Correlation	.647	.680*	-.298	.953**	.741*	.851**	1	.848**	.946**	.928**	.697*	.919**	.767*	.789*	.732*	.703*	.902**	.118	.667*	.861**	.444	.743*
	Sig. (2-tailed)	.060	.044	.436	<.001	.022	.004		.004	<.001	<.001	.037	<.001	.016	.011	.025	.035	<.001	.763	.049	.003	.232	.022
	N	9	9	9	9	9	9	9	9	9	9	9	9	9	9	9	9	9	9	9	9	9	9
Lys	Pearson Correlation	.209	.266	-.059	.764*	.960**	.979**	.848**	1	.967**	.610	.930**	.978**	.981**	.961**	.956**	.890**	.677*	.563	.927**	.469	.769*	.778*
	Sig. (2-tailed)	.589	.489	.880	.017	<.001	<.001	.004		<.001	.081	<.001	<.001	<.001	<.001	<.001	.001	.045	.114	<.001	.203	.016	.014
	N	9	9	9	9	9	9	9	9	9	9	9	9	9	9	9	9	9	9	9	9	9	9
Met	Pearson Correlation	.426	.463	-.186	.881**	.909**	.955**	.946**	.967**	1	.774*	.849**	.986**	.924**	.910**	.881**	.832**	.802**	.398	.849**	.664	.665	.807**
	Sig. (2-tailed)	.253	.209	.632	.002	<.001	<.001	<.001	<.001		.014	.004	<.001	<.001	<.001	.002	.005	.009	.289	.004	.051	.051	.008
	N	9	9	9	9	9	9	9	9	9	9	9	9	9	9	9	9	9	9	9	9	9	9
Phe	Pearson Correlation	.857**	.792*	-.285	.915**	.479	.598	.928**	.610	.774*	1	.388	.712*	.508	.568	.440	.428	.951**	-.182	.385	.971**	.164	.677*
	Sig. (2-tailed)	.003	.011	.457	<.001	.192	.089	<.001	.081	.014		.302	.031	.163	.111	.237	.250	<.001	.639	.306	<.001	.674	.045
	N	9	9	9	9	9	9	9	9	9	9	9	9	9	9	9	9	9	9	9	9	9	9
Thr	Pearson Correlation	-.020	.188	-.122	.608	.889**	.962**	.697*	.930**	.849**	.388	1	.910**	.927**	.848**	.980**	.903**	.406	.645	.887**	.283	.723*	.517
	Sig. (2-tailed)	.960	.628	.755	.082	.001	<.001	.037	<.001	.004	.302		<.001	<.001	.004	<.001	<.001	.278	.060	.001	.460	.028	.154
	N	9	9	9	9	9	9	9	9	9	9	9	9	9	9	9	9	9	9	9	9	9	9
Val	Pearson Correlation	.328	.434	-.175	.840**	.917**	.986**	.919**	.978**	.986**	.712*	.910**	1	.944**	.918**	.936**	.887**	.732*	.463	.881**	.602	.693*	.736*
	Sig. (2-tailed)	.390	.243	.652	.005	<.001	<.001	<.001	<.001	<.001	.031	<.001		<.001	<.001	<.001	.001	.025	.209	.002	.086	.038	.024
	N	9	9	9	9	9	9	9	9	9	9	9	9	9	9	9	9	9	9	9	9	9	9
Ala	Pearson Correlation	.110	.129	.062	.650	.983**	.956**	.767*	.981**	.924**	.508	.927**	.944**	1	.944**	.969**	.883**	.608	.700*	.961**	.358	.843**	.781*
	Sig. (2-tailed)	.777	.741	.874	.058	<.001	<.001	.016	<.001	<.001	.163	<.001	<.001		<.001	<.001	.002	.083	.036	<.001	.344	.004	.013
	N	9	9	9	9	9	9	9	9	9	9	9	9	9	9	9	9	9	9	9	9	9	9
Arg	Pearson Correlation	.117	.141	.074	.698*	.925**	.905**	.789*	.961**	.910**	.568	.848**	.918**	.944**	1	.899**	.895**	.695*	.531	.938**	.391	.826**	.823**
	Sig. (2-tailed)	.765	.718	.851	.037	<.001	<.001	.011	<.001	<.001	.111	.004	<.001	<.001		<.001	.001	.038	.141	<.001	.298	.006	.006
	N	9	9	9	9	9	9	9	9	9	9	9	9	9	9	9	9	9	9	9	9	9	9
Asp	Pearson Correlation	.022	.158	-.042	.608	.941**	.972**	.732*	.956**	.881**	.440	.980**	.936**	.969**	.899**	1	.909**	.493	.698*	.934**	.310	.798**	.617

	Sig. (2-tailed)	.955	.684	.914	.083	<.001	<.001	.025	<.001	.002	.237	<.001	<.001	<.001	<.001		<.001	.177	.037	<.001	.417	.010	.077
	N	9	9	9	9	9	9	9	9	9	9	9	9	9	9	9	9	9	9	9	9	9	9
Glu	Pearson Correlation	-.071	.179	-.065	.627	.847**	.906**	.703*	.890**	.832**	.428	.903**	.887**	.883**	.895**	.909**	1	.490	.540	.937**	.312	.816**	.586
	Sig. (2-tailed)	.855	.646	.869	.071	.004	<.001	.035	.001	.005	.250	<.001	.001	.002	.001	<.001		.181	.134	<.001	.414	.007	.097
	N	9	9	9	9	9	9	9	9	9	9	9	9	9	9	9	9	9	9	9	9	9	9
Gly	Pearson Correlation	.748*	.572	-.045	.848**	.587	.613	.902**	.677*	.802**	.951**	.406	.732*	.608	.695*	.493	.490	1	-.029	.518	.865**	.353	.852**
	Sig. (2-tailed)	.020	.108	.909	.004	.097	.079	<.001	.045	.009	<.001	.278	.025	.083	.038	.177	.181		.942	.153	.003	.351	.004
	N	9	9	9	9	9	9	9	9	9	9	9	9	9	9	9	9	9	9	9	9	9	9
Pro	Pearson Correlation	-.428	-.458	.372	-.041	.715*	.549	.118	.563	.398	-.182	.645	.463	.700*	.531	.698*	.540	-.029	1	.723*	-.322	.790*	.373
	Sig. (2-tailed)	.250	.215	.324	.917	.030	.126	.763	.114	.289	.639	.060	.209	.036	.141	.037	.134	.942		.028	.398	.011	.323
	N	9	9	9	9	9	9	9	9	9	9	9	9	9	9	9	9	9	9	9	9	9	9
Ser	Pearson Correlation	-.073	-.008	.093	.539	.958**	.901**	.667*	.927**	.849**	.385	.887**	.881**	.961**	.938**	.934**	.937**	.518	.723*	1	.230	.942**	.742*
	Sig. (2-tailed)	.852	.983	.811	.134	<.001	<.001	.049	<.001	.004	.306	.001	.002	<.001	<.001	<.001	<.001	.153	.028		.552	<.001	.022
	N	9	9	9	9	9	9	9	9	9	9	9	9	9	9	9	9	9	9	9	9	9	9
Tyr	Pearson Correlation	.903**	.873**	-.396	.865**	.327	.487	.861**	.469	.664	.971**	.283	.602	.358	.391	.310	.312	.865**	-.322	.230	1	-.004	.525
	Sig. (2-tailed)	<.001	.002	.291	.003	.390	.183	.003	.203	.051	<.001	.460	.086	.344	.298	.417	.414	.003	.398	.552		.992	.147
	N	9	9	9	9	9	9	9	9	9	9	9	9	9	9	9	9	9	9	9	9	9	9
Asn	Pearson Correlation	-.262	-.272	.187	.282	.881**	.725*	.444	.769*	.665	.164	.723*	.693*	.843**	.826**	.798**	.816**	.353	.790*	.942**	-.004	1	.680*
	Sig. (2-tailed)	.497	.479	.629	.462	.002	.027	.232	.016	.051	.674	.028	.038	.004	.006	.010	.007	.351	.011	<.001	.992		.044
	N	9	9	9	9	9	9	9	9	9	9	9	9	9	9	9	9	9	9	9	9	9	9
Gln	Pearson Correlation	.415	.130	.242	.646	.790*	.660	.743*	.778*	.807**	.677*	.517	.736*	.781*	.823**	.617	.586	.852**	.373	.742*	.525	.680*	1
	Sig. (2-tailed)	.267	.740	.531	.060	.011	.053	.022	.014	.008	.045	.154	.024	.013	.006	.077	.097	.004	.323	.022	.147	.044	
	N	9	9	9	9	9	9	9	9	9	9	9	9	9	9	9	9	9	9	9	9	9	9

**. Correlation is significant at the 0.01 level (2-tailed).

*. Correlation is significant at the 0.05 level (2-tailed).

Confidence Intervals

	Pearson Correlation	Sig. (2-tailed)	95% Confidence Intervals (2-tailed)[a]	
			Lower	Upper
FRAP - ORAC	.838	<.001	.551	.942
FRAP - DPP_IV	-.327	.235	-.713	.234
FRAP - DH	.619	.014	.135	.853
FRAP - His	.102	.793	-.607	.715
FRAP - Ile	.202	.602	-.543	.758
FRAP - Leu	.647	.060	-.071	.910
FRAP - Lys	.209	.589	-.538	.761
FRAP - Met	.426	.253	-.356	.842
FRAP - Phe	.857	.003	.404	.966
FRAP - Thr	-.020	.960	-.674	.654
FRAP - Val	.328	.390	-.447	.807
FRAP - Ala	.110	.777	-.602	.718
FRAP - Arg	.117	.765	-.598	.721
FRAP - Asp	.022	.955	-.652	.676
FRAP - Glu	-.071	.855	-.700	.625
FRAP - Gly	.748	.020	.121	.938
FRAP - Pro	-.428	.250	-.843	.353
FRAP - Ser	-.073	.852	-.701	.624
FRAP - Tyr	.903	<.001	.558	.977
FRAP - Asn	-.262	.497	-.782	.500

FRAP - Gln	.415	.267	-.366	.838
ORAC - DPP_IV	-.549	.034	-.822	-.031
ORAC - DH	.686	.005	.245	.881
ORAC - His	.086	.826	-.617	.707
ORAC - Ile	.352	.353	-.426	.816
ORAC - Leu	.680	.044	-.014	.920
ORAC - Lys	.266	.489	-.496	.784
ORAC - Met	.463	.209	-.316	.855
ORAC - Phe	.792	.011	.224	.950
ORAC - Thr	.188	.628	-.552	.752
ORAC - Val	.434	.243	-.347	.845
ORAC - Ala	.129	.741	-.590	.727
ORAC - Arg	.141	.718	-.583	.732
ORAC - Asp	.158	.684	-.572	.740
ORAC - Glu	.179	.646	-.559	.748
ORAC - Gly	.572	.108	-.184	.888
ORAC - Pro	-.458	.215	-.853	.322
ORAC - Ser	-.008	.983	-.668	.660
ORAC - Tyr	.873	.002	.456	.970
ORAC - Asn	-.272	.479	-.787	.491
ORAC - Gln	.130	.740	-.590	.727
DPP_IV - DH	-.357	.191	-.729	.202
DPP_IV - His	-.006	.988	-.667	.661
DPP_IV - Ile	-.193	.618	-.755	.549
DPP_IV - Leu	-.298	.436	-.796	.471

DPP_IV - Lys	-.059	.880	-.694	.632
DPP_IV - Met	-.186	.632	-.751	.554
DPP_IV - Phe	-.285	.457	-.792	.481
DPP_IV - Thr	-.122	.755	-.724	.595
DPP_IV - Val	-.175	.652	-.747	.561
DPP_IV - Ala	.062	.874	-.630	.695
DPP_IV - Arg	.074	.851	-.624	.701
DPP_IV - Asp	-.042	.914	-.686	.641
DPP_IV - Glu	-.065	.869	-.697	.629
DPP_IV - Gly	-.045	.909	-.687	.640
DPP_IV - Pro	.372	.324	-.407	.824
DPP_IV - Ser	.093	.811	-.612	.710
DPP_IV - Tyr	-.396	.291	-.832	.385
DPP_IV - Asn	.187	.629	-.553	.752
DPP_IV - Gln	.242	.531	-.514	.775
DH - His	.612	.080	-.126	.900
DH - Ile	.766	.016	.162	.943
DH - Leu	.953	<.001	.763	.989
DH - Lys	.764	.017	.156	.942
DH - Met	.881	.002	.483	.972
DH - Phe	.915	<.001	.603	.980
DH - Thr	.608	.082	-.131	.899
DH - Val	.840	.005	.353	.962
DH - Ala	.650	.058	-.066	.911
DH - Arg	.698	.037	.019	.924
DH - Asp	.608	.083	-.132	.899

DH - Glu	.627	.071	-.102	.905
DH - Gly	.848	.004	.377	.964
DH - Pro	-.041	.917	-.685	.642
DH - Ser	.539	.134	-.227	.879
DH - Tyr	.865	.003	.429	.968
DH - Asn	.282	.462	-.483	.791
DH - Gln	.646	.060	-.071	.910
His - Ile	.933	<.001	.675	.984
His - Leu	.741	.022	.106	.936
His - Lys	.960	<.001	.794	.991
His - Met	.909	<.001	.581	.979
His - Phe	.479	.192	-.298	.860
His - Thr	.889	.001	.509	.974
His - Val	.917	<.001	.612	.981
His - Ala	.983	<.001	.911	.996
His - Arg	.925	<.001	.643	.982
His - Asp	.941	<.001	.712	.986
His - Glu	.847	.004	.374	.964
His - Gly	.587	.097	-.162	.893
His - Pro	.715	.030	.053	.929
His - Ser	.958	<.001	.785	.990
His - Tyr	.327	.390	-.447	.807
His - Asn	.881	.002	.481	.972
His - Gln	.790	.011	.218	.949
Ile - Leu	.851	.004	.386	.965
Ile - Lys	.979	<.001	.887	.995

Ile - Met	.955	<.001	.773	.990
Ile - Phe	.598	.089	-.147	.896
Ile - Thr	.962	<.001	.805	.991
Ile - Val	.986	<.001	.924	.997
Ile - Ala	.956	<.001	.775	.990
Ile - Arg	.905	<.001	.568	.978
Ile - Asp	.972	<.001	.855	.994
Ile - Glu	.906	<.001	.569	.978
Ile - Gly	.613	.079	-.124	.901
Ile - Pro	.549	.126	-.214	.882
Ile - Ser	.901	<.001	.550	.977
Ile - Tyr	.487	.183	-.290	.862
Ile - Asn	.725	.027	.072	.932
Ile - Gln	.660	.053	-.049	.914
Leu - Lys	.848	.004	.377	.964
Leu - Met	.946	<.001	.732	.987
Leu - Phe	.928	<.001	.657	.983
Leu - Thr	.697	.037	.018	.924
Leu - Val	.919	<.001	.619	.981
Leu - Ala	.767	.016	.164	.943
Leu - Arg	.789	.011	.216	.949
Leu - Asp	.732	.025	.086	.934
Leu - Glu	.703	.035	.029	.926
Leu - Gly	.902	<.001	.554	.977
Leu - Pro	.118	.763	-.597	.722
Leu - Ser	.667	.049	-.036	.916

Leu - Tyr	.861	.003	.416	.967
Leu - Asn	.444	.232	-.337	.848
Leu - Gln	.743	.022	.110	.937
Lys - Met	.967	<.001	.830	.992
Lys - Phe	.610	.081	-.129	.900
Lys - Thr	.930	<.001	.663	.984
Lys - Val	.978	<.001	.882	.995
Lys - Ala	.981	<.001	.899	.996
Lys - Arg	.961	<.001	.799	.991
Lys - Asp	.956	<.001	.775	.990
Lys - Glu	.890	.001	.513	.974
Lys - Gly	.677	.045	-.018	.919
Lys - Pro	.563	.114	-.195	.886
Lys - Ser	.927	<.001	.651	.983
Lys - Tyr	.469	.203	-.310	.856
Lys - Asn	.769	.016	.167	.943
Lys - Gln	.778	.014	.189	.946
Met - Phe	.774	.014	.180	.945
Met - Thr	.849	.004	.380	.964
Met - Val	.986	<.001	.924	.997
Met - Ala	.924	<.001	.639	.982
Met - Arg	.910	<.001	.584	.979
Met - Asp	.881	.002	.481	.972
Met - Glu	.832	.005	.331	.960
Met - Gly	.802	.009	.249	.952
Met - Pro	.398	.289	-.383	.833

Met - Ser	.849	.004	.381	.964
Met - Tyr	.664	.051	-.042	.915
Met - Asn	.665	.051	-.040	.915
Met - Gln	.807	.008	.263	.954
Phe - Thr	.388	.302	-.393	.829
Phe - Val	.712	.031	.047	.929
Phe - Ala	.508	.163	-.266	.869
Phe - Arg	.568	.111	-.189	.887
Phe - Asp	.440	.237	-.342	.847
Phe - Glu	.428	.250	-.353	.843
Phe - Gly	.951	<.001	.752	.989
Phe - Pro	-.182	.639	-.750	.556
Phe - Ser	.385	.306	-.395	.828
Phe - Tyr	.971	<.001	.847	.993
Phe - Asn	.164	.674	-.568	.742
Phe - Gln	.677	.045	-.019	.919
Thr - Val	.910	<.001	.584	.979
Thr - Ala	.927	<.001	.651	.983
Thr - Arg	.848	.004	.377	.964
Thr - Asp	.980	<.001	.892	.995
Thr - Glu	.903	<.001	.558	.977
Thr - Gly	.406	.278	-.375	.835
Thr - Pro	.645	.060	-.073	.910
Thr - Ser	.887	.001	.502	.973
Thr - Tyr	.283	.460	-.483	.791
Thr - Asn	.723	.028	.068	.931

Thr - Gln	.517	.154	-.254	.872
Val - Ala	.944	<.001	.723	.987
Val - Arg	.918	<.001	.615	.981
Val - Asp	.936	<.001	.688	.985
Val - Glu	.887	.001	.503	.973
Val - Gly	.732	.025	.087	.934
Val - Pro	.463	.209	-.317	.854
Val - Ser	.881	.002	.482	.972
Val - Tyr	.602	.086	-.141	.897
Val - Asn	.693	.038	.010	.923
Val - Gln	.736	.024	.095	.935
Ala - Arg	.944	<.001	.724	.987
Ala - Asp	.969	<.001	.836	.993
Ala - Glu	.883	.002	.489	.972
Ala - Gly	.608	.083	-.132	.899
Ala - Pro	.700	.036	.024	.925
Ala - Ser	.961	<.001	.800	.991
Ala - Tyr	.358	.344	-.420	.819
Ala - Asn	.843	.004	.361	.962
Ala - Gln	.781	.013	.197	.947
Arg - Asp	.899	<.001	.546	.976
Arg - Glu	.895	.001	.530	.975
Arg - Gly	.695	.038	.014	.924
Arg - Pro	.531	.141	-.237	.876
Arg - Ser	.938	<.001	.696	.986
Arg - Tyr	.391	.298	-.390	.830

Arg - Asn	.826	.006	.312	.958
Arg - Gln	.823	.006	.304	.957
Asp - Glu	.909	<.001	.581	.979
Asp - Gly	.493	.177	-.282	.864
Asp - Pro	.698	.037	.020	.925
Asp - Ser	.934	<.001	.679	.985
Asp - Tyr	.310	.417	-.461	.801
Asp - Asn	.798	.010	.238	.951
Asp - Gln	.617	.077	-.118	.902
Glu - Gly	.490	.181	-.287	.863
Glu - Pro	.540	.134	-.226	.879
Glu - Ser	.937	<.001	.695	.985
Glu - Tyr	.312	.414	-.460	.802
Glu - Asn	.816	.007	.286	.956
Glu - Gln	.586	.097	-.164	.893
Gly - Pro	-.029	.942	-.679	.649
Gly - Ser	.518	.153	-.253	.872
Gly - Tyr	.865	.003	.428	.968
Gly - Asn	.353	.351	-.425	.817
Gly - Gln	.852	.004	.388	.965
Pro - Ser	.723	.028	.069	.931
Pro - Tyr	-.322	.398	-.805	.451
Pro - Asn	.790	.011	.218	.949
Pro - Gln	.373	.323	-.407	.824
Ser - Tyr	.230	.552	-.523	.770
Ser - Asn	.942	<.001	.714	.986

Ser - Gln	.742	.022	.108	.937
Tyr - Asn	-.004	.992	-.666	.662
Tyr - Gln	.525	.147	-.245	.874
Asn - Gln	.680	.044	-.014	.920

a. Estimation is based on Fisher's r-to-z transformation with bias adjustment.
