# Supplementary material for: In Vitro Gastrointestinal Digestion of Calanus finmarchicus Products: Amino Acid Composition, Degree of Hydrolysis, Antioxidant Capacity, and Antidiabetic Activity
Source: Mar Drugs. 2026 Jul 7;24(7):240. doi: 10.3390/md24070240 (PMC13412531; doi:10.3390/md24070240)
Supplement: Supplementary file 1 [file marinedrugs-24-00240-s001.zip › Table_S3_FDCF_PTP1B-and-TC-PTP-Inhibitory-Activity.pdf]

Table S3. Protein tyrosine phosphatase 1B (PTP1B) inhibitory activity and T-cell protein tyrosine phosphatase (TC-PTP) counter-screen for freeze-dried *C. finmarchicus* (FDCF) during *in vitro* gastrointestinal digestion at 0, 30, 75, 105, and 165 min. Each time point includes three independent digestions (n = 3).

Sample	Digestion time (min)	PTP1B inhibitory activity (%)	PTP1B status[1]	TC-PTP inhibitory activity (%)[2]	TC-PTP status[1]
FDCF	0	95	Inactive	n.m.	n.m.
FDCF	0	116	Inactive	n.m.	n.m.
FDCF	0	66	Inactive	n.m.	n.m.
FDCF	30	76	Inactive	n.m.	n.m.
FDCF	30	86	Inactive	n.m.	n.m.
FDCF	30	82	Inactive	n.m.	n.m.
FDCF	75	91	Inactive	n.m.	n.m.
FDCF	75	84	Inactive	n.m.	n.m.
FDCF	75	83	Inactive	n.m.	n.m.
FDCF	105	69	Inactive	n.m.	n.m.
FDCF	105	73	Inactive	n.m.	n.m.
FDCF	105	129	Inactive	n.m.	n.m.
FDCF	165	17	Active	21	Active
FDCF	165	147	Inactive	n.m.	n.m.
FDCF	165	44	Questionable	n.m.	n.m.

[1]Status thresholds applied to activity: Active < 30%; Questionable 30-40%; Inactive > 40%. Negative or > 100% can occur due to baseline correction. [2]TC-PTP was measured only for replicates classified as Active for PTP1B. n.m. = not measured.
