# Supplementary material for: In Vitro Gastrointestinal Digestion of Calanus finmarchicus Products: Amino Acid Composition, Degree of Hydrolysis, Antioxidant Capacity, and Antidiabetic Activity
Source: Mar Drugs. 2026 Jul 7;24(7):240. doi: 10.3390/md24070240 (PMC13412531; doi:10.3390/md24070240)
Supplement: Supplementary file 1 [file marinedrugs-24-00240-s001.zip › Table_S2_FFCF_PTP1B-and-TC-PTP-Inhibitory-Activity.pdf]

Table S2. Protein tyrosine phosphatase 1B (PTP1B) inhibitory activity and T-cell protein tyrosine phosphatase (TC-PTP) counter-screen for fresh-frozen *C. finmarchicus* (FFCF) during *in vitro* gastrointestinal digestion at 0, 30, 75, 105, and 165 min. Each time point includes three independent digestions ($n = 3$).

Sample	Digestion time (min)	PTP1B inhibitory activity (%)	PTP1B status[1]	TC-PTP inhibitory activity (%)[2]	TC-PTP status[1]
FFCF	0	-2	Active	-8	Active
FFCF	0	-7	Active	28	Active
FFCF	0	-6	Active	26	Active
FFCF	30	-3	Active	-7	Active
FFCF	30	-7	Active	20	Active
FFCF	30	-9	Active	13	Active
FFCF	75	108	Inactive	n.m.	n.m.
FFCF	75	97	Inactive	n.m.	n.m.
FFCF	75	100	Inactive	n.m.	n.m.
FFCF	105	32	Question	122	Inactive
FFCF	105	73	Inactive	n.m.	n.m.
FFCF	105	87	Inactive	n.m.	n.m.
FFCF	165	45	Inactive	n.m.	n.m.
FFCF	165	51	Inactive	n.m.	n.m.
FFCF	165	70	Inactive	n.m.	n.m.

[1]Status thresholds applied to activity: Active < 30%; Questionable 30-40%; Inactive > 40%. Negative or > 100% can occur due to baseline correction. [2]TC-PTP was measured only for replicates classified as Active for PTP1B. n.m. = not measured.
